# Prevalence of ocular and visual abnormalities following symptomatic and asymptomatic congenital CMV infection: a systematic review and meta-analysis

**DOI:** 10.1016/j.eclinm.2025.103443

**Published:** 2025-09-02

**Authors:** Tamar Schreiber, Naomi Tan, Alice Bellchambers, Sohaib R. Rufai, Nutifafa Thywill Adorkor, Umar Ahmed, Harry Petrushkin, Ameenat Lola Solebo

**Affiliations:** a Population, Policy and Practice Department of Research and Teaching, UCL GOS Institute of Child Health, London, UK; b University College London Medical School, London, UK; c Department of Ophthalmology, Royal Free London NHS Foundation Trust, United Kingdom; d University Hospitals Sussex NHS Foundation Trust, Sussex, UK; e University of Leicester Ulverscroft Eye Unit, Leicester, UK; f Clinical and Academic Department of Ophthalmology, Great Ormond Street Hospital for Children NHS Trust, London, UK; g Imperial Hospital Medical School, London, UK; h Department of Ophthalmology, East Sussex Healthcare NHS Trust, Sussex, UK; i Moorfields Eye Hospital, London, UK; j National Institute for Health Research Biomedical Research Centre at Moorfields Eye Hospital NHS Foundation Trust and UCL Institute of Ophthalmology, London, UK; k National Institute for Health Research Biomedical Research Centre at Great Ormond Street Hospital and UCL GOS ICH, London, UK

**Keywords:** Congenital cytomegalovirus, Visual impairment, Eye disorder

## Abstract

**Background:**

Cytomegalovirus (CMV), the most common congenitally acquired infection, can result in visual disability in affected children. We aimed to estimate the burden of eye and vision disorders amongst children with symptomatic and asymptomatic congenital cytomegalovirus infection (cCMV), to inform the development of guidance for the provision of care.

**Methods:**

In this systematic review and meta-analysis, we searched PubMed, Embase, and CINAHL databases up to 6th Feb 2025 for studies reporting ocular disorders or visual impairment (VI) outcomes following cCMV diagnosis. We included longitudinal or cross-sectional studies which reported the frequency of visual or ophthalmic outcomes following an initial diagnosis of symptomatic or asymptomatic cCMV. Summary data, and individual patient level data where available, on the proportions of children noted to have visual impairment or ophthalmic disorders and the manifestation of these disorders, were extracted from published reports. Pooled prevalence of eye and vision outcomes were estimated through random effects models computed using Restricted Maximum Likelihood (REML) estimation. We included studies at lower risk of bias (assessed using the Joanna Briggs Institute tool) in meta-analyses of prevalence (random-effect models) and undertook subgroup analyses. The review protocol was registered with PROSPERO, CRD42021284678.

**Findings:**

We identified 4488 articles of which 28 were eligible for inclusion. Of these, 15 studies (total 858 children with symptomatic, 1176 with asymptomatic cCMV) were eligible for meta-analyses. Median follow up time from diagnosis of cCMV ranged from 6 to 156 months. Estimated pooled prevalence in symptomatic cCMV of visual impairment (VI) and ocular disorders 9% (95% CI, 5–14%, *I*^*2*^ = 51.09%) and 14% (95% confidence interval, CI, 5–31%, *I*^*2*^ = 93.2%) respectively. Cerebral visual impairment (i.e. VI due to neurological insult rather than ocular disease) was the most commonly reported visual disability, with an estimated pooled prevalence of 10% (95% CI, 6–15%, *I*^*2*^ = 24.9). Prevalence of ocular disorders (most commonly chorioretinitis, and optic nerve and anterior segment anomalies) was higher in studies with greater proportions of pre-term birth, hearing impairment, and those undertaken prior to 2017. Estimated pooled prevalence of VI and ocular disorders was 1% and <1% (95% CI, 0–2%, *I*^2^ = 0%) respectively in asymptomatic cCMV.

**Interpretation:**

Visual disability in cCMV is a strong marker of the broader neurological insult. Ocular disorders are prevalent in symptomatic disease, with consequent need for ongoing ophthalmic care. The low prevalence of sight-impactful disorders in asymptomatic disease suggests little benefit for ongoing ophthalmic surveillance, particularly in health settings with established programmes for whole population childhood eye and vision screening. This review is limited by the absence of information on the timing of diagnosis of the eye and vision disorders, data which would support the development of timeline pathways for ophthalmic surveillance.

**Funding:**

10.13039/100010269Wellcome Trust, 10.13039/501100000272National Institute for Health and Care Research (NIHR).

**Research in context**

**Evidence before this study**

A preliminary search of PubMed, Web of Science database, and Google Scholar, which scoped the existing evidence on visually impactful complications of congenital cytomegalovirus (cCMV) infection between January 1, 1970, and February 1, 2025, with no restriction by language, and with search terms including “eyes OR vision” AND “congenital cytomegalovirus”, identified a number of studies on visual and ophthalmic outcomes in cCMV. A systematic review published in 2023 included 17 studies and reported on the frequency of eye disorders in cCMV, but did not differentiate prevalence in symptomatic versus asymptomatic disease, and did not undertake meta-analyses.

**Added value of this study**

Our review included 28 articles published between 1977 and 2025 in order to describe the prevalence of ocular and or visual disorders following symptomatic and asymptomatic cCMV. We used internationally accepted terminology for eye disorders and to categorise visual impairment. Our meta-analyses provide pooled estimates which suggest that one in ten children with symptomatic cCMV will grow up with visual impairment or blindness, and more will have structural ocular anomalies or disorders which require ongoing ophthalmic care, and which put them at risk of later life sight loss. In the majority of reported cases, childhood visual disability in cCMV was due to neurological rather than ocular causes. Our analyses also suggest a low likelihood of visual disorders in children with asymptomatic cCMV diagnosed following the introduction of the 2017 consensus based international diagnostic guidelines. Co-occurring pre-term birth, and later confirmation of sensorineural hearing loss or neurodevelopmental impairment may increase the risk of sight and ocular disorder in children with initially asymptomatic cCMV.

**Implications of all the available evidence**

For children with symptomatic cCMV, follow up assessment of visual function during the first few years of life is important, as intervention and support during this developmental phase are key for good outcomes for children with or at risk of poor vision. This is particularly important as the burden of dual sensory impairment (hearing and sight loss) in symptomatic cCMV is unclear but likely to be significant. As children with asymptomatic cCMV can be considered to have similar eye health care needs to the general population of children, there is little need for additional surveillance, but it is important that families are counselled about the importance of the whole population or ‘healthy child’ eye health checks and screening programmes for their child. This review is limited by the absence of information on the timing of detection or onset of new eye and vision disorders following an initial diagnosis of cCMV, with this data being essential for the development of ophthalmic surveillance pathways.

## Introduction

Congenitally acquired infections are responsible for a significant proportion of the global burden of infant mortality and morbidity.^1^^,^^2^ The most commonly acquired congenital infection is cytomegalovirus (CMV), affecting an estimated 1%–2% of live births worldwide.^3^ Up to 1 in 5 neonates with congenital CMV (cCMV) will be symptomatic,^4^^,^^5^ with manifestations including disorders affecting the eyes and cerebral visual pathways (Box 1).^6^, ^7^, ^8^ Thus, visual disability is a recognised sequelae of congenital cytomegalovirus (cCMV) infection, and improved understanding about the ocular and visual impact of cCMV should enable the development of care processes for children at risk.

Box 1 **Consensus based definition of symptomatic congenital cytomegalovirus infection6**

**Table:** 

**Neonates**	
Physical examination	Hepatosplenomegaly Neonatal petechiae/purpura/rash Jaundice Microcephaly^a^/small for gestational age
Laboratory parameters	Prolonged/Conjugated hyperbilirubinemia Unexplained thrombocytopenia/leukopenia/anaemia
Neurology & Neuroimaging	Seizures^a^ Intracranial calcification^a^/ventriculomegaly^a^ and structural anomalies^a^
Visual examination	Chorioretinitis^a^, cataracts^a^, structural anomalies^a^
Maternal serology	Evidence of maternal seroconversion
Prematurity	
Failed neonatal hearing screen	
**Older children**	
New diagnosis of sensorineural hearing loss	

a Potentially visually disabling disorders.

Childhood onset visual disability has a strikingly negative impact on broader developmental, educational and quality of life outcomes.^9^^,^^10^ These negative outcomes can be mitigated either by timely intervention for amenable disorders (such as congenital cataract) or timely visual habilitation to address the needs of those with irreversibly poor vision (i.e. formal developmental, educational and mobility support).^8^^,^^11^

The typical neonate's visual acuity level would be declared as ‘legally blind’ if present in an adult: worse than 1.0 on the logarithm of Minimal Angle of Resolution scale, logMAR, i.e. worse than the largest letter on a standard vision chart.^8^ In the normally developing child, visual acuity improves dramatically over the first few years of life. This developmental trajectory may be delayed in infants with neurological disorders.^12^ Consequently, the negative impact of cCMV on visual function, and the specific developmental support and visual habilitation needs for the affected child may be uncertain until nearer the developmental stage at which ‘normal’ levels of acuity tend to emerge (3–5 years old).^13^ Although the recent European Congenital Infection Initiative concluded that “ophthalmological follow-up is only recommended for those infants with retinitis at birth and not required for newborns with normal retinal examination”,^6^^,^^7^ there is a lack of consensus on eye and vision care needs for the broader population of children with cCMV.

We aim to provide the evidence needed to develop recommendations to support the provision of care needs for children with congenital cytomegalovirus (CMV), by estimating the prevalence of eye or vision related disorders following a diagnosis of asymptomatic or symptomatic cCMV.

## Methods

### Search strategy and selection criteria

We used the PRISMA (Preferred Reporting Items for Systematic review and Meta-Analysis) recommendations for this systematic review.^14^^,^^15^ The full details of the methods used have been reported elsewhere,^16^ but are summarised below. This review was prospectively registered (PROSPERO, registration number CRD42021284678). Screening and data collection were undertaken using Covidence (Veritas Health Innovation, Australia, www.covidence.org). We searched databases from inception to 6th Feb 2025 (full search strategy in Supplementary document) in order to identify eligible studies. Inclusion criteria comprised a study population of patients with congenital cytomegalovirus (cCMV) diagnosed by urine or blood, and study design which enabled the reporting of the frequency of visual or ophthalmic outcomes following the initial diagnosis of symptomatic or asymptomatic cCMV. Title, abstracts and then full texts were screened by at least two co-authors. Full text screeners also manually reviewed references cited within eligible articles to identify additional potentially eligible studies. Discordant screening results were resolved by discussion between the two screeners. In the event of a failure to reach consensus, abstracts were included for full text review. Following full text review, where there was disagreement between reviewers on inclusion, the final decision was made by the senior author (ALS).

A study specific form (modified Covidence template, Supplemental Data) was used to extract data from eligible full texts. Independent double extraction was undertaken. The International Classification of Disease (ICD-11) definitions of visual impairment and blindness were used to categorise visual disability (moderate visual impairment being vision worse than 0.48 logMAR, and severe visual impairment/blindness being vision worse than 1.0 logMAR).^17^ Ocular disorders were defined using the categorisations developed for childhood blinding ocular disorders.^18^ Specifically, structural ocular disorders (disordered development of the globe, i.e. not including eye disorders such as strabismus or amblyopia) were categorised as affecting the retina/choroid, the optic nerve, or the anterior segment. Where multiple studies reported ocular and visual outcomes from the same dataset or cohort, we excluded duplicate study with the smallest sample size or the shortest follow-up duration, unless different outcomes were reported across the different articles.

We used a Joanna Briggs Institute (JBI) tool to assess methodological quality of included articles.^19^ Studies were classified as having a low, moderate or high risk of bias on the basis of the overall score, and consequently judged to be of high, moderate or low methodological quality respectively.

### Data analysis

All analyses and derivation of forest plots were done in Stata 18.5 (StataCorp, College Station, TX). Data on the proportions of children noted to have visual impairment or ophthalmic disorders, and the manifestation of these disorders, were initially analysed descriptively. Separate pooled prevalence of visual impairment, and of structural ocular disorders, was estimated through random effects models computed using Restricted Maximum Likelihood (REML) estimation in conjunction with the Freeman-Tukey transformation to stabilise the variance of proportions. Meta-analysis was limited to those studies with low or moderate scores on risk of bias assessment. We used separate random-effects models to pool prevalence amongst patients with symptomatic and asymptomatic cCMV. We estimated heterogeneity between studies using Cochran's Q (p < 0.05 indicating moderate heterogeneity) and *I*^2^ statistics (≥50% or higher indicating moderate heterogeneity). Sensitivity of pooled estimates to individual studies was examined using Leave-One-Out analysis.^20^ Subgroup analyses were undertaken, using the following covariates: study period (pre versus post 2017, i.e. the date of the development of the European diagnostic criteria for symptomatic cCMV),^6^ study design (retrospective versus prospective), follow up time (more or less than 5 years), study population (population with hearing loss versus ‘whole’ population with cCMV, and high rate of preterm birth within study population versus low rate, with a threshold set at 10%), country income (using Organisation for Economic Co-operation and Development rankings of low, middle or high), and methodological quality assessment score (moderate versus low risk of bias). Publication bias was evaluated using Funnel plots and Egger's tests. The confidence in the pooled prevalence was quantified using a modified GRADE (Grading of Recommendations, Assessment, Development and Evaluations) four level scale, running from ‘high’ (very confident that the true frequency lies close to that of the estimate, with low heterogeneity, non-significant Q scores, heterogeneity captured by sub-group analyses, and no evidence of under-reporting of insignificant results on publication bias analyses) to ‘very low’ (very little confidence: the true frequency is likely to be substantially different from the estimate).

### Role of the funding source

The funder of the study had no role in study design, data collection, data analysis, data interpretation, or writing of the report. All authors had full access to all the data in the study, and had final responsibility for the decision to submit for publication.

## Results

**Fig. 1:**
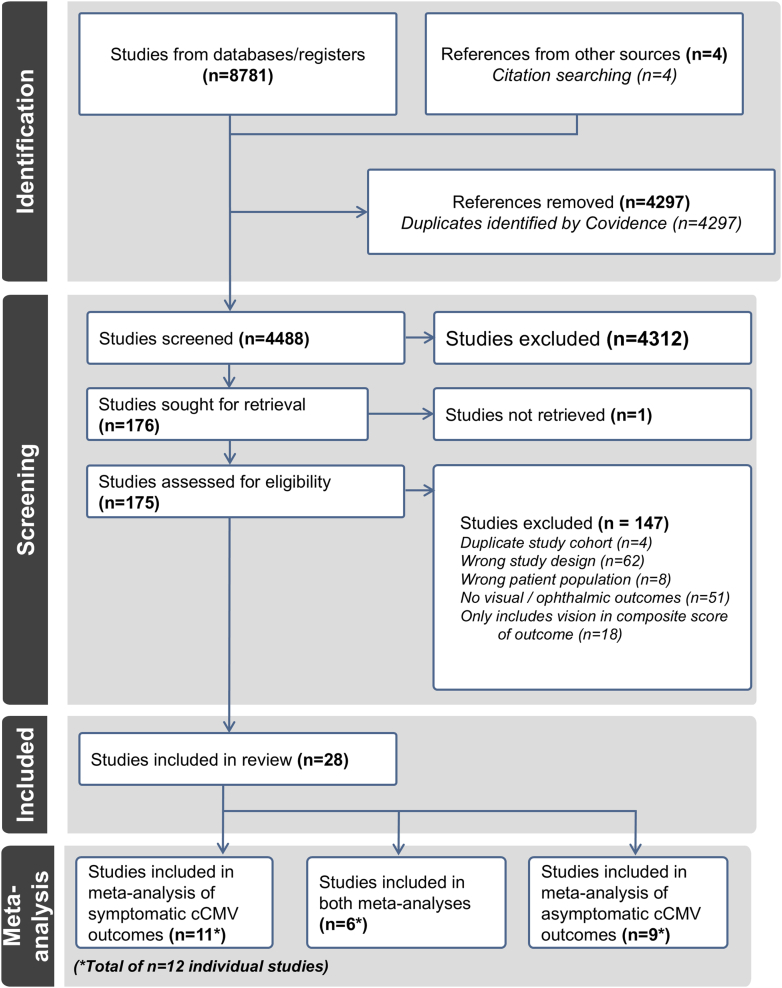
PRISMA flow diagram.

We identified 4488 individual articles through database and manual searching (Fig. 1). Following screening, 176 articles were selected for full-text review, with 28 of the 176 articles deemed eligible for inclusion in the systematic review. Amongst included studies, 27 articles reported ocular and/or visual outcomes for a total of 1059 individual children with symptomatic cCMV,^21^, ^22^, ^23^, ^24^, ^25^, ^26^, ^27^, ^28^, ^29^, ^30^, ^31^, ^32^, ^33^, ^34^, ^35^, ^36^, ^37^, ^38^, ^39^, ^40^, ^41^, ^42^, ^43^, ^44^, ^45^, ^46^, ^47^, ^48^ and 18 articles reported outcomes following an initial diagnosis of asymptomatic cCMV in 1432 children.^21^^,^^22^^,^^24^^,^^26^, ^27^, ^28^, ^29^, ^30^, ^31^, ^32^, ^33^, ^34^, ^35^^,^^37^^,^^39^, ^40^, ^41^, ^42^ Three studies reported different outcomes from the same study cohort.^24^^,^^25^^,^^37^ Characteristics of the studies are detailed in Table 1.

**Table 1:** Characteristics of included studies.

Study ID	Median follow up (months)	Minimum follow up (months)	Region	Study setting	Study design	Dates	Criteria for participant inclusion in the study	Symptomatic CMV	Asymptomatic cCMV	Ethnicity	Proportion female	Proportion preterm birth
Alarcon 2013^20^	104	NP	Europe	Single Secondary + Setting	Retrospective cross -sectional study	1993–2006	1. Viral DNA in urine or blood, or CMV IgM/viral antigen in blood during the first 2 weeks of life AND 2. Symptomatic^a^ cCMV	0	24	NP	46%	35%
Auriti 2022^21^^,^^b^	NP	24	Europe	Single Secondary + Setting	Retrospective cohort study	2011–2020	1. Viral DNA in urine/saliva/blood, or CMV IgM in blood during the first 3 weeks	29	55	NP	NP	0%
Capretti 2017^22^^,^^b^	35	12	Europe	Single Secondary + Setting	Prospective cohort study	2006–2015	1. Viral DNA in urine, or on DBS in first 3 weeks, or viral copy load in blood in first 3 weeks	30	18	NP	46%	NP
Dreher 2014^23^	NP	NP	North America	Single Secondary + Setting	Retrospective cohort study	1980–2002	1. Viral DNA in urine/saliva during the first 3 weeks AND 2. Symptomatic^a^ cCMV	0	166	White: Black: Hispanic 86:91:1 49%:50%: <1%	45%	27%
Engman 2008^24^^,^^b^	NP	36	Europe	Multicentre Secondary + Settings	Retrospective cohort study	2003–2004	1. DNA positive DBS samples at 3–5 days confirmed by viral DNA in urine/saliva/blood	11	0	Swedish: Non-Swedish 8:3 72%:28%	NP	27%
Engman 2010^25^	NP	NP	Europe	Single Secondary + Setting	Retrospective and prospective cohort study	1998–2008	1. DNA positive DBS samples at 3–5 days confirmed by viral DNA in urine/saliva/blood AND 2. Neurological disabilities and or cerebral cortical malformations detected by MRI or CT	0	4	NP	NP	0%
Forner 2015^26^	NP	12	Europe	Single Secondary + Setting	Prospective cohort study	2004–2007	1. Maternal CMV IgG and IgM positivity AND 2. Viral DNA in urine/blood during the first 3 weeks	33	0	NP	NP	0%
Fukushima 2019^27^^,^^b^	NP	18	Asia	Single Secondary + Setting	Prospective cohort study	2009–2018	1. Viral DNA in urine/saliva during the first 3 weeks AND 2. Symptoms: at least 1 of microcephaly, small for gestational age (SGA), hepatitis, structural brain anomalies on MRI, ocular complications, hearing impairment AND 3. Treated with oral Valganciclovir	0	21	Japanese	76%	NP
Jin 2017^28^^,^^a^^,^^b^	132	NP	North America	Single Secondary + Setting	Prospective cohort study	1982–1992	1. Viral DNA in urine in first 3 weeks AND 2. Symptoms: at least one of SGA, generalized petechial rash, hepatomegaly, splenomegaly, jaundice, microcephaly, seizures, thrombocytopenia	109	77	White non-Hispanic: White Hispanic: African American: Asian 128:29:27:2 69%:16%:15:1%	48%	NP
Jin 2019^29^^,^^a^^,^^b^	132	NP	North America	Single Secondary + Setting	Retrospective cohort study	1982–1992	1. Viral DNA in urine in first 3 weeks AND 2. Symptoms: at least one of SGA, generalized petechial rash, hepatomegaly, splenomegaly, jaundice, microcephaly, seizures, thrombocytopenia	11	66	White (Non-Hispanic): Hispanic: African American: Asian 58:19:12:2 64%:21%:13%:2%	53%	31%
Karimian 2016^30^	NP	12	Middle East	Multicentre Secondary + Settings	Prospective cohort study	2014–2016	1. Viral DNA in urine during the first week AND 2. Symptomatic^a^ cCMV	5	3	NP	63%	25%
Karltorp 2014^31^	94	10	Europe	Single Secondary + Setting	Prospective cohort study	2002–2012	1. Cochlear implant AND 2. Hearing impairment of previously unknown AND 3. DNA positive DBS samples at 3–5 days	20	6	NP	54%	15%
Keymeulen 2023^32^^,^^b^	72	6	Europe	Multicentre Secondary + Settings	Prospective cohort study	2007–2020	1. Viral DNA in urine/saliva/blood, or CMV IgM in DBS in the first 3 weeks	261	492	NP	48%	8%
Korndewal 2017^33^	NP	72	Europe	Whole population	Retrospective Cohort study	2008	1. DNA positive DBS samples at 1–5 days AND 2. Confirmatory viral DNA in blood	107	26	NP	44%	10%
Kylat 2006^34^^,^^b^	NP	24	North America	Unclear	Retrospective cohort study	1987–2000	1. Viral DNA in urine/saliva/secretions during the first 3 weeks AND 2. Symptoms: hearing impairment, petechiae, hepatosplenomegaly, jaundice, microcephaly, hydrocephaly, other congenital anomalies, motor abnormalities, SGA, prematurity, chorioretinitis	0	42	Caucasian: African American: Other 20:8:12 50%: 20%: 30%	60%	21%
Lanzieri 2017^35^	156	NP	North America	Multicentre Secondary + Settings	Retrospective cohort study	1983–2005	1. Viral DNA in urine during the first 3 weeks AND 2. Symptomatic, with at least 1 of: purpura/petechiae, jaundice, hepatosplenomegaly, microcephaly, unexplained neurological abnormality, elevated alanine aminotransferase/or total bilirubin, hemolytic anemia, or thrombocytopenia	0	76	White (Non-Hispanic): Other 43:33 57%:43%	54%	32%
Lin 2020^36^	NP	6	Asia	Whole population	Retrospective cross-sectional study	2010–2017	1. At least 1 diagnosis coded as cCMV (ICD-10 code P35.1) within first month of life	17	36	NP	45%	NP
Marin 2016^37^	NP	24	South America	Multicentre Secondary + Settings	Prospective cohort study	2010–2012	1. Viral DNA in urine/saliva in first 3 weeks	24	1	NP	NP	4%
Pass 1980^38^	42	NP	Europe	Single Secondary + Setting	Prospective cohort study	1965–1979	1. Viral DNA in urine during the first 3 weeks AND 2. Symptoms: petechiae, hepatosplenomegaly, jaundice, microcephaly, hydrocephaly, other congenital anomalies, SGA, prematurity, or chorioretinitis	0	23	White: Non-white 28/34:6/34 82%:18%	44%	NP
Puhakka 2019^39^^,^^b^	NP	18	Europe	Multicentre Secondary + Settings	Prospective cohort study	2012–2015	1. Viral DNA in urine during the first week AND 2. Viral DNA in urine/saliva at 3 months of age	34	1	NP	NP	0%
Salomè 2023^b^	84	NP	Europe	Single Secondary + Setting	Prospective cohort study	2002	1. Maternal CMV IgG and IgM positivity OR Symptoms^a^ suggestive of cCMV AND 2. Viral DNA in urine/blood during the first 3 weeks	127	123	NP	48%	NP
Stagno 1977^40^	NP	21	North America	Single Secondary + Setting	Prospective cohort study	Pre 1977	1. Maternal CMV IgG (de novo appearance) during pregnancy AND 2. Viral DNA in urine during the first week	35	8	NP	NP	NP
Tear Fahnehjelm 2015^41^^,^^b^	96	16	Europe	Whole population	Retrospective and prospective cohort study	2002–2012	1. Cochlear implant AND 2. Hearing impairment of previously unknown aetiology AND 3. CMV IgM/viral antigen in blood AND 4. DNA positive DBS samples at 3–5 days	20	6	NP	54%	NP
Townsend 2013^42^^,^^b^	NP	60	Europe	Multinational Secondary + Settings	Prospective cohort study	1977–1985	1. Viral DNA in urine during the first 2 weeks AND 2. Maternal sera CMV IgG and IgM positive	157	19	White: Black: Asian 147:22:7 84%:13%:4%	49%	6%
Turriziani Colonna 2020^43^	50	NP	Europe	Single Secondary + Setting	Retrospective cohort study	2009–2017	1. Viral DNA in urine/saliva during the first 3 weeks AND 2. Treated with oral Valganciclovir	24	12	NP	NP	6%

DBS: dried blood spot.

DNA: Deoxyribonucleic acid.

Ig: Immunoglobulin.

SGA: small for gestational age.

NP: not provided.

Secondary+: secondary care and higher (tertiary/quaternary care).

a Definition consistent with European Expert Consensus Statement definition of symptomatic disease.

b Included in meta-analyses.

Studies were conducted across higher (n = 26) and middle income (n = 2) settings, specifically USA (n = 8), Italy, (n = 5), Sweden (n = 5), Japan, (n = 2), Belgium, Croatia, Finland, Netherlands, Spain and the United Kingdom/UK, n = 1 each), and Brazil and Iran (n = 1 each). One study was multinational (UK and Sweden). Study populations ranged from 44% to 76% female, and prevalence of pre-term birth (less than 37 weeks gestational age) ranged from 0 to 35%. Median follow up time ranged from 6 to 156 months from diagnosis of cCMV, whilst minimum duration of follow up ranged from 4 months to 18 years. A range of assessments were used to capture visual and ocular outcomes (Supplementary Document, Figure S1). The most common assessment was fundus examination (specified as undertaken for the whole cohort in 15 of the 28 studies). In 11 studies (39%) there were no detailed descriptions of the ocular or visual assessments undertaken.

Methodological quality was judged to be good (low overall risk of bias on JBI tool assessment) for 6 articles, moderate for 9 and poor for 12 (high overall risk of bias) (Supplemental Document, Figure S2). Thus, 15 studies, reporting outcomes for a total 858 individual children with symptomatic and 1176 with asymptomatic cCMV, were used in the meta-analyses. The most common methodological concerns were small sample sizes, incomplete reporting of outcomes across the whole study sample, and absence of reported use of validated or repeatable methods of assessing visual and or ocular outcomes.

**Fig. 2:**
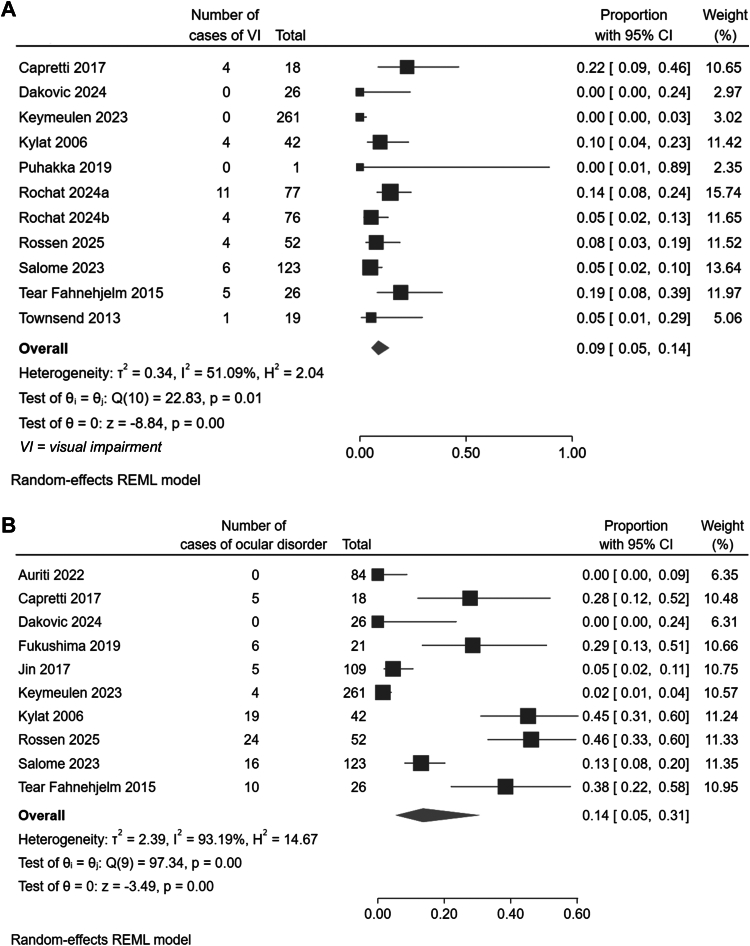
Forest plot showing the proportion of children with symptomatic cCMV found to have (A) visual impairment and (B) structural ocular anomalies or congenital disorders at follow up.

The proportion of children with visual impairment (VI) following a diagnosis of symptomatic cCMV ranged from 0% to 22% across all studies. Pooled prevalence of all cause visual impairment, using only those studies judged to be of good or moderate quality, was estimated at 9% (95% confidence interval, CI, 5–14%, Cochran's Q (10) = 22.83, p < 0.01, and *I*^2^ = 51.09%, confidence in pooled prevalence moderate) (Fig. 2A). Subgroup analyses did not identify significant differences in prevalence associated with cCMV diagnostic modality, completeness of reported ophthalmic assessment, study setting, design, population or quality, or length of follow up as sources of the heterogeneity seen (subgroup tests of group difference in Supplementary Document, Table S1). Cases of cerebral visual impairment, as reported across five studies, affected between 0 and 14% of study populations, and were responsible for the majority of cases of reported visual disability for which a ‘cause’ was reported (Supplementary Document, Table S2). The estimated overall pooled prevalence of CVI (cortical or cerebral visual impairment) in symptomatic cCMV was 10% (95% CI, 6–15%, Cochran's Q (3) = 3.98, p = 0.26, and *I*^2^ = 24.85%, confidence in pooled prevalence moderate, Supplementary Figure S3).

**Fig. 3:**
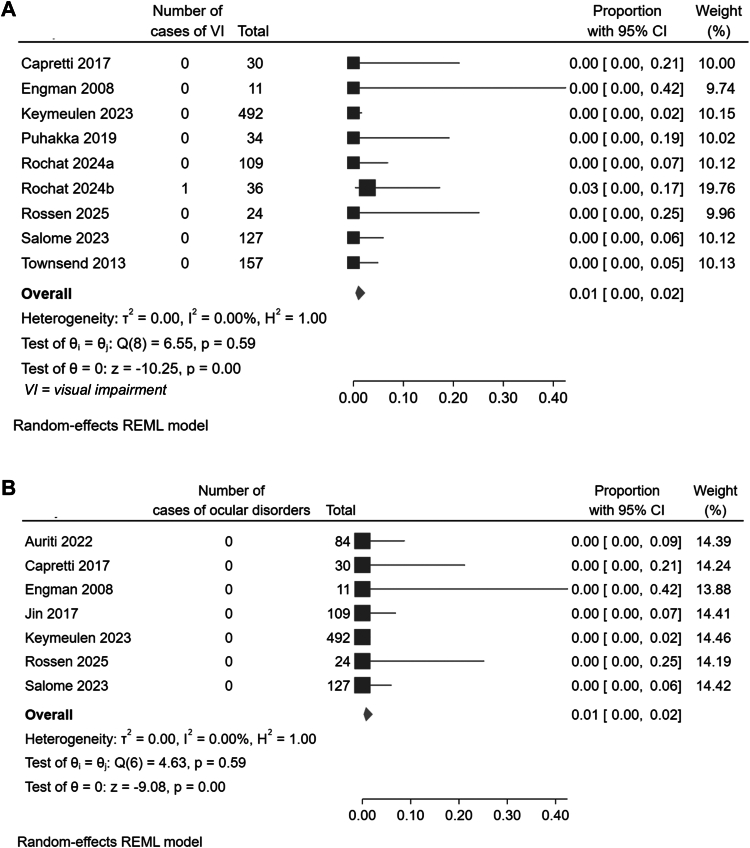
Forest plot showing the proportion of children with asymptomatic cCMV found to have (A) visual impairment and (B) structural ocular anomalies or congenital disorders at follow up.

Following an initial diagnosis of asymptomatic cCMV, the prevalence of VI across all studies ranged from 0 to 3%, with a pooled prevalence estimated at 1% (95% CI, 0–2%, Cochran's Q (8) = 6.55, p = 0.60 and *I*^2^ = 0%, confidence in pooled prevalence high) (Fig. 3A), and no evidence of subgroup differences (Supplementary Document, Table S3).

The proportion of children with ocular structural or congenital disorders following a diagnosis of symptomatic cCMV ranged from 0% to 50% across all studies. Pooled prevalence overall was estimated at 14% (95% confidence interval, CI, 5–31%, Cochran's Q (10) = 97.34, p < 0.01, and *I*^2^ = 93.19%, confidence in pooled prevalence low) (Fig. 2B). Prevalence estimates were greater amongst populations of children with symptomatic cCMV identified due to hearing impairment (affecting 38%, 95% CI, 22–58%|, versus 13%, 95% CI, 5–29% across the whole population of children with symptomatic cCMV, test of group differences: Qb (1) = 4.86, p = 0.03, Supplementary Table S4), amongst studies undertaken prior to 2017 (affecting 43%, 95% CI, 34–52%, versus 10%, 95% CI, 3–24% of children in studies undertaken at a later date, Qb (1) = 11.32, p < 0.001), higher amongst populations with a higher burden of pre-term birth (38%, 95% CI, 24–53%, versus 2%, 95% CI, 1–4% amongst those studies with lower proportions of participants with pre-term birth, Qb (1) = 37.65, p < 0.001) and higher amongst those studies with shorter follow up durations (37%, 95% CI, 29–47% across studies with less than 5 years follow up, versus 7%, 95% CI, 2–19%, Qb (1) = 11.20, p < 0.001).

The most commonly occurring ocular structural or congenital disorders were chorioretinitis (pooled prevalence 11%, 95% CI, 5–23%, Cochran's Q (7) = 44.9, p < 0.001, and *I*^2^ = 86.45%, confidence in pooled prevalence low, Forest plot provided in Supplemental Document Figure S4); optic nerve hypoplasia or anomalies (pooled prevalence 6%, 95% CI, 3–14%, Cochran's Q (6) = 20.64, p = 0.05, and *I*^2^ = 71.05%, confidence in pooled prevalence low, Supplementary Figure S5) and anterior segment anomalies such as congenital cataract and congenital corneal opacity (pooled prevalence 6%, 95% CI, 3–9%, Cochran's Q (4) = 5.33, p = 0.26, and *I*^2^ = 0%, confidence in pooled prevalence high, Supplementary Figure S6). A full list of identified ocular disorders (all forms) and frequencies of occurrence are presented in the supplementary documentation (Supplementary Tables S5 and S6).

Following a diagnosis of asymptomatic cCMV, the prevalence of ocular structural or congenital disorders across all studies, irrespective of study quality, ranged from 0 to 10%. When limited to the studies which were appropriate for inclusion in meta-analyses (i.e. studies graded as good or moderate quality), prevalence across those studies ranged from 0%–3%. Pooled prevalence was estimated at <1%, 95% CI, 0–2%, Cochran's Q (8) = 6.55, p = 0.59, and *I*^2^ = 0%, confidence in pooled prevalence high) (Fig. 3B). Subgroup analyses were not undertaken due to a prevalence of 0% in all but one of the included studies.

Sensitivity analyses revealed that the pooled prevalence for ocular and visual disorders following asymptomatic cCMV were robust to omission of individual studies on Leave-One-Out analyses (Supplementary Figures S6). The estimated pooled prevalences of ocular and visual disorders following symptomatic cCMV were reasonably robust, with omission of the largest study cohort resulting in slightly higher pooled prevalence of both ocular disorders and visual impairment. Although interpretation of the funnel plots was limited by the small number of studies,^49^ plot asymmetries suggestive of publication bias and or reflective of study heterogeneity were identified (Supplemental Data, Figure S7). Regression-based Egger tests suggested a small-study effect for the pooled prevalence of ocular disorders (with smaller studies reporting higher prevalence) in symptomatic cCMV (beta 1 −3.62, z −2.11, p = 0.03).

## Discussion

In this systematic review and meta-analysis, we present estimates of the prevalence of eye and vision disorders following a diagnosis of cCMV. There was variability of prevalence across study populations, possibly driven by differences in the classification of symptomatic disease over time, and by study population prevalences of sensorineural healing loss (SNHL) and pre-term birth. Chorioretinal lesions, optic nerve anomalies or anterior segment dysgenesis were the most commonly identified eye disorders. For those papers which reported causes, the majority of cases of VI were due to cerebral insult. There were no cases of eye anomalies following a diagnosis of asymptomatic cCMV across studies with patients diagnosed following the introduction of the international consensus-based taxonomy for symptomatic cCMV.

Long term neurodevelopmental impairments have been reported to occur in half of all children with symptomatic cCMV, versus 14% of cases amongst those with initially asymptomatic disease, with disabilities typically limited to later development of sensorineural hearing loss.^5^^,^^50^, ^51^, ^52^ The prevalence of visual problems (including common childhood disorders such as refractive error) has been estimated at 16.3% for children with cCMV,^53^ irrespective of symptomatic status. Visual disability (i.e. visual impairment defined using WHO criteria)^54^ in children with symptomatic cCMV is less common, previously estimated at 6%, and 3% in asymptomatic cCMV.^55^ The lower prevalence of visual disability in symptomatic cCMV and higher prevalence in asymptomatic cCMV reported by this older review may reflect historic under-ascertainment of symptomatic cCMV. This hypothesis is indirectly supported by the impact of study period on the pooled prevalence of sight impactful ocular disorders as identified through our subgroup analyses.^6^

The introduction of international guidance on the diagnostic criteria of cCMV has been accompanied by improvements in neuroimaging,^56^ and serological testing.^57^ Despite evidence of remaining variability in the definition of symptomatic cCMV in use across the literature,^50^ and ongoing limitations in cCMV testing,^50^^,^^51^ these advancements may have resulted in larger proportions of children with milder phenotypes being correctly diagnosed as symptomatic, which might result in an apparent reduction of morbidity in the ‘asymptomatic’ group. This will have implications for systematic reviews of other developmental outcomes following cCMV. However, there has been no similar reduction in hearing impairment: up to 10% of children with initially asymptomatic cCMV develop hearing loss, and this figure has stayed stable over time.^5^^,^^28^^,^^32^^,^^52^ Again, improved detection of disease may play a part: differing diagnostics and decibel thresholds have been used over time to diagnose SNHL.^58^

The impact of follow up duration on pooled prevalence reported here may also be an indicator of the burden of cCMV: it can be hypothesised that studies with a longer follow up are more likely to be those in which outcomes have been assessed at a later age.^58^^,^^59^ The population who have survived to this later age are less likely to have had the severe manifestations of symptomatic cCMV, and thus are less likely to have had the congenital anomalies more associated with poor survival.^59^ Visual impairment is known to be associated with a four times higher mortality rate amongst affected infants.^18^ It is likely that visual impairment or ocular disorders in children with cCMV acts as a predictive marker of poorer overall health outcomes.

CMV is the most common congenitally acquired congenital infection, with a fast growing incidence, and a differential distribution amongst and within countries, with those in the most under-resourced socioeconomic strata being most likely to bear the burden of disease.^60^^,^^61^ Potential approaches to this population health crisis include family education on preventive strategies such as hand washing, maternal testing for primary CMV infection during the first trimester of pregnancy and or universal newborn screening (to allow prompt case detection, necessary for improved outcome), improved test modalities to identify reactivation of disease in women already infected, anti-viral treatment for those mothers testing positive, and the development and implementation of an effective CMV vaccine.^61^, ^62^, ^63^, ^64^, ^65^ Measurement of the impact of these future strategies will require an understanding of the existing burden of cCMV.^65^ The data presented here should be useful benchmarking for the impact of future interventions on outcomes across the population of all affected infants, and across vulnerable subgroups such as those with hearing loss or those born pre-term.

Preventing some or all of the negative impact of or early onset childhood sight loss involves primary preventive methods (preventing the blinding disease from occurring), secondary prevention (early detection of the eye disease to minimise the risk of blindness) or tertiary prevention of the burden of blindness (preventing the negative impact of established sight loss).^8^ Whole population eye health preventive approaches are embedded into several public health systems,^11^ for example indirect testing of visual function through assessment of broader motor and co-ordination skills, as for example undertaken within the UK at age 2 years within the healthy child programme.^66^ The UK and other nations also undertake eye screening for all neonates and infants, and vision screening for all children aged 4–5 years old, to enable timely detection of ocular anomalies or poor vision respectively.^67^ Families of children with cCMV should be kept aware of these ‘whole population’ healthy child programmes within their child's health setting.

**Fig. 4:**
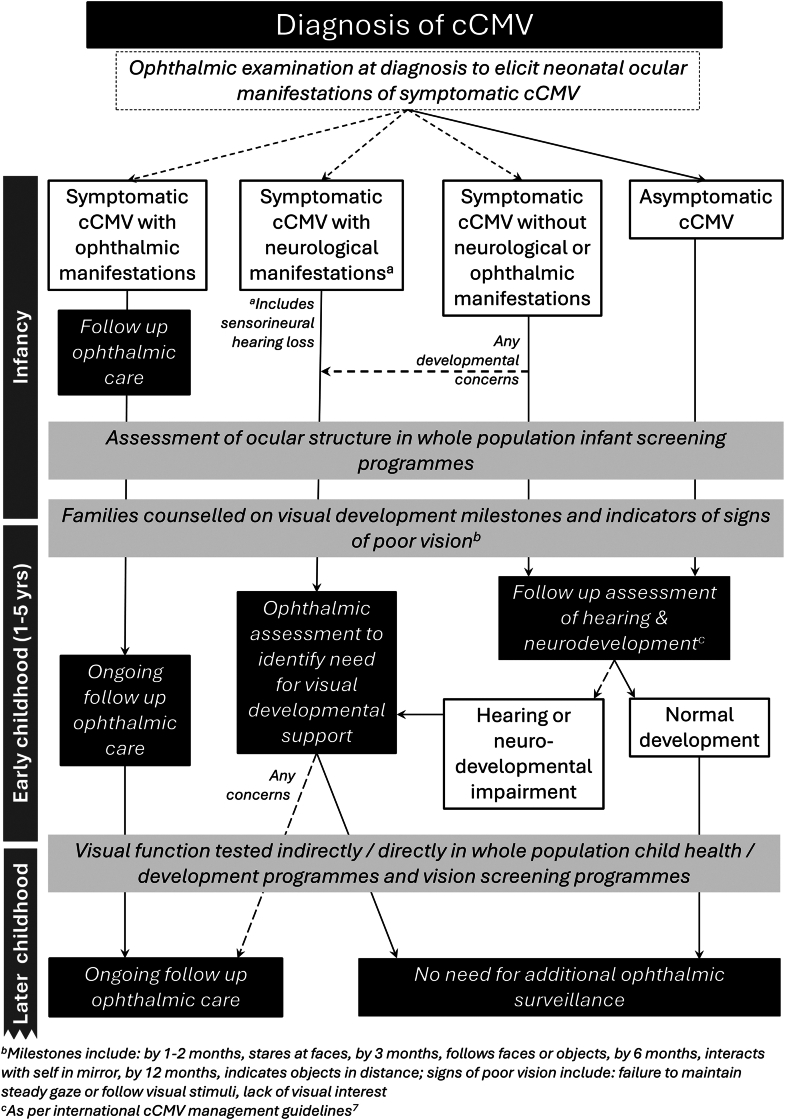
Congenital CMV ocular and visual follow up care algorithm.

In addition to whole population approaches, targeted surveillance of children at risk is also available across many health care settings.^11^^,^^66^^,^^68^ The current guidance that “ophthalmological follow-up is not required for newborns {with cCMV} with normal retinal examination”^7^ omits the other eye disorders which may impact this population. Even in the absence of a known ocular disorder, infants with neurodevelopmental disorders (irrespective of cause) should be considered to be at risk of vision impairment (Fig. 4). There are higher rates of impactful visual developmental disorders in this group^69^^,^^70^ and a need for intervention during the developmental period of neuroplasticity.^71^ In some cases, neurodevelopmental concerns (typically sensorineural hearing loss) may develop in children who were initially categorised as having asymptomatic cCMV. These children will then also require targeted ophthalmic surveillance (Fig. 4). For those children with asymptomatic cCMV, or for those whose symptoms have not resulted in neurodevelopmental, hearing or ocular disorders, the low prevalence of sight threating ocular disorders supports the absence of need for ophthalmic surveillance beyond the whole population public health interventions available within that health care setting.

The absence of information on the timing of diagnosis of the eye and vision disorders limits the use of our findings in supporting the development of structured, timeline pathways of ophthalmic surveillance. Our findings do, however indicate key milestones. Structural ocular or congenital eye disorders present at birth would ideally be excluded through ophthalmic examination in the neonatal phase, removing the need for continued ophthalmic surveillance. Detection of these congenital ocular disorders can be challenging, particularly in children with additional health care needs, and managing ophthalmologists may need to schedule re-examination.^72^ Disorders of visual function may only be apparent once the child has reached a developmental stage at which full uniocular assessments can be undertaken, necessitating later examination of the child identified to be at risk. Thus, the pooled prevalences reported here are valuable indicators of shape of ongoing need for ophthalmic assessment after diagnosis of cCMV. Our focus on ocular anomalies (i.e. those which confer a risk of blindness) resulted in an exclusion of cases of strabismus, and of refractive errors such as astigmatism from the pooled prevalence estimates. These eye disorders are commonly occurring in up to 5% of healthy children, and are thus target disorders for ‘whole population’ health programmes,^67^ rather than a focus for surveillance in the high risk cCMV population. We have however reported summary frequencies in Supplementary Table S5.

The small number of studies eligible for meta-analyses limits the interpretation of the funnel plots. Study heterogeneity across those studies reporting outcomes for symptomatic cCMV is suggested. However, quantification of heterogeneity enables some exploration of the impact of those study differences. Our reports of higher prevalence of eye and visual disorders in certain groups, e.g., those born pre-term supports the appraisal and interpretation of existing studies and supports the design of future studies of outcome. There are groups of children who have not been represented within these meta-analyses: those living in low and middle income countries are at the greatest risk of cCMV infection, and of poor outcomes.^3^^,^^60^ The pooled prevalence estimates presented here may not generalise to those populations, particularly as co-occurrent potentially blinding disorders such as other congenital infections, pre term birth and perinatal ischaemic insult confer additional and potentially synergistic risks on children in these under resourced areas.^8^

In conclusion, we present robust estimates of the burden of ocular and visual sequelae following cCMV in asymptomatic, as well as symptomatic children. The burden of cerebral visual impairment, and of dual sensory impairment (hearing and sight loss) in symptomatic cCMV is unclear but likely to be significant. For children presenting with neurodevelopmental concerns following diagnosis of symptomatic cCMV, it remains important to conduct follow-up assessment of visual function during early childhood, to ensure timely intervention and support during this developmentally sensitive time.^6^^,^^7^^,^^62^ comparable to those of the general population, reducing the necessity for additional surveillance. However, families of these children should be counselled about the importance of eye health and population-based “healthy child” vision screening programs. Of these children should be counselled about the importance of routine eye health assessments and participation in population-based “healthy child” vision screening programs.

## Contributors

ALS conceptualised the study, developed the methodology, and undertook data collection, analysis, supervision, drafting and final approval and guarantor of review. TS contributed to data collection, analysis and manuscript drafting. AB contributed to data collection, and critical revision. FA contributed to data collection and analysis. UA, SRR, NT, WT and HP contributed to methodology, data collection and critical revision of the manuscript. All authors had full access to all the data in the study, and had final responsibility for the decision to submit for publication. ALS, TS, FA and AB directly accessed and verified the underlying data reported in the manuscript.

## Data sharing statement

No additional data are available. Data for this study were extracted from the published literature. The dataset supporting the conclusions of this article is included within the Article and its appendix.

## Declaration of interests

ALS has received: consultancy fees from Alimera Sciences Ltd and speaker honoraria from Heidelberg Engineering for work unrelated to the manuscript; grants from Fight for Sight and Medical Research Foundation; and support for attending meetings from the European Alliance of Associations for Rheumatology and the Royal College of Ophthalmologists. There are no other competing interests to declare.

## References

[1] Madrid L., Varo R., Sitoe A., Bassat Q. (2016). Congenital and perinatally-acquired infections in resource-constrained settings. Expert Rev Anti Infect Ther.

[2] Zhang L., Wang X., Liu M. (2022). The epidemiology and disease burden of congenital TORCH infections among hospitalized children in China: a national cross-sectional study. PLoS Negl Trop Dis.

[3] Ssentongo P., Hehnly C., Birungi P. (2021). Congenital cytomegalovirus infection burden and epidemiologic risk factors in countries with universal screening. JAMA Netw Open.

[4] Pesch M.H., Lauer C.S., Weinberg J.B. (2024). Neurodevelopmental outcomes of children with congenital cytomegalovirus: a systematic scoping review. Pediatr Res.

[5] Dollard S.C., Grosse S.D., Ross D.S. (2007). New estimates of the prevalence of neurological and sensory sequelae and mortality associated with congenital cytomegalovirus infection. Rev Med Virol.

[6] Rawlinson W.D., Boppana S.B., Fowler K.B. (2017). Congenital cytomegalovirus infection in pregnancy and the neonate: consensus recommendations for prevention, diagnosis, and therapy. Lancet Infect Dis.

[7] Leruez-Ville M., Chatzakis C., Lilleri D. (2024). Consensus recommendation for prenatal, neonatal and postnatal management of congenital cytomegalovirus infection from the European congenital infection initiative (ECCI). Lancet Reg Health Eur.

[8] Solebo A.L., Teoh L., Rahi J. (2017). Epidemiology of blindness in children. Arch Dis Child.

[9] Cumberland P.M., Rahi J.S., UK Biobank Eye and Vision Consortium (2016). Visual function, social position, and health and life chances: the UK biobank study. JAMA Ophthalmol.

[10] Rahi J.S., Cumberland P.M., Peckham C.S. (2009). Visual impairment and vision-related quality of life in working-age adults: findings in the 1958 British birth cohort. Ophthalmology.

[11] Keel S., Lingham G., Misra N. (2022). Toward universal eye health coverage-key outcomes of the world health organization package of eye care interventions: a systematic review. JAMA Ophthalmol.

[12] Tresidder J., Fielder A.R., Nicholson J. (1990). Delayed visual maturation: ophthalmic and neurodevelopmental aspects. Dev Med Child Neurol.

[13] Ellemberg D., Lewis T.L., Hong Liu C., Maurer D. (1999). Development of spatial and temporal vision during childhood. Vision Res.

[14] Stroup D.F., Berlin J.A., Morton S.C. (2000). Meta-analysis of observational studies in epidemiology: a proposal for reporting. Meta-analysis of observational studies in epidemiology (MOOSE) group. JAMA.

[15] Moher D., Shamseer L., Clarke M. (2015). Preferred reporting items for systematic review and meta-analysis protocols (PRISMA-P) 2015 statement. Syst Rev.

[16] Karamchandani U., Ahmed U., Rufai S.R. (2022). Long-term ocular and visual outcomes following symptomatic and asymptomatic congenital CMV infection: a systematic review protocol. BMJ Open.

[17] Kv V., Vijayalakshmi P. (2020). Understanding definitions of visual impairment and functional vision. Community Eye Health.

[18] Teoh L.J., Solebo A.L., Rahi J.S. (2021). Visual impairment, severe visual impairment, and blindness in children in britain (BCVIS2): a national observational study. Lancet Child Adolesc Health.

[19] Moola S., Munn Z., Sears K. (2015). Conducting systematic reviews of association (etiology). Int J Evid Based Healthc.

[20] Higgins J.P.T. (2008). Commentary: heterogeneity in meta-analysis should be expected and appropriately quantified. Int J Epidemiol.

[21] Auriti C., Bucci S., De Rose D.U. (2022). Maternal–fetal infections (cytomegalovirus, toxoplasma, syphilis): short-term and long-term neurodevelopmental outcomes in children infected and uninfected at birth. Pathogens.

[22] Capretti M.G., Marsico C., Guidelli Guidi S. (2017). Neonatal and long-term ophthalmological findings in infants with symptomatic and asymptomatic congenital cytomegalovirus infection. J Clin Virol.

[23] Engman M., Lewensohn-Fuchs I., Mosskin M., Malm G. (2010). Congenital cytomegalovirus infection: the impact of cerebral cortical malformations. Acta Paediatr.

[24] Jin H.D., Demmler-Harrison G.J., Coats D.K. (2017). Long-term visual and ocular sequelae in patients with congenital cytomegalovirus infection. Pediatr Infect Dis J.

[25] Jin H.D., Demmler-Harrison G.J., Miller J. (2019). Cortical visual impairment in congenital cytomegalovirus infection. J Pediatr Ophthalmol Strabismus.

[26] Karimian P., Yaghini O., Nasr Azadani H. (2016). Prevalence, characteristics, and one-year follow-up of congenital cytomegalovirus infection in Isfahan city, Iran. Interdiscip Perspect Infect Dis.

[27] Karltorp E., Löfkvist U., Lewensohn-Fuchs I. (2014). Impaired balance and neurodevelopmental disabilities among children with congenital cytomegalovirus infection. Acta Paediatr.

[28] Keymeulen A., De Leenheer E., Casaer A. (2023). Neurodevelopmental outcome in children with congenital cytomegalovirus infection: a prospective multicenter cohort study. Early Hum Dev.

[29] Korndewal M.J., Oudesluys-Murphy A.M., Kroes A.C.M., van der Sande M.A.B., de Melker H.E., Vossen A.C.T.M. (2017). Long-term impairment attributable to congenital cytomegalovirus infection: a retrospective cohort study. Dev Med Child Neurol.

[30] Lin C., Tomio J., Tanaka H., Sonoda M., Sano K., Kobayashi Y. (2020). Diagnosis and medical care for congenital cytomegalovirus infection. Medicine.

[31] Marin L.J., Santos de Carvalho Cardoso E., Bispo Sousa S.M. (2016). Prevalence and clinical aspects of CMV congenital infection in a low-income population. Virol J.

[32] Puhakka L., Lappalainen M., Lönnqvist T. (2019). The burden of congenital cytomegalovirus infection: a prospective cohort study of 20,000 infants in Finland. J Pediatric Infect Dis Soc.

[33] Salomè S., Ciampa N., Giordano M. (2023). Ophthalmological impairment in patients with congenital cytomegalovirus infection. Front Pediatr.

[34] Stagno S. (1986). Primary cytomegalovirus infection in pregnancy. JAMA.

[35] Teär Fahnehjelm K., Olsson M., Fahnehjelm C., Lewensohn-Fuchs I., Karltorp E. (2015). Chorioretinal scars and visual deprivation are common in children with cochlear implants after congenital cytomegalovirus infection. Acta Paediatr.

[36] Đaković I., Kostović I., Vulin K. (2024). Are important predictors of adverse outcome in children with symptomatic congenital cytomegalovirus infection overlooked in clinical settings?. J Int Med Res.

[37] Rochat R., Goodman E., Miller J., Wang W., Demmler-Harrison G.J. (2024). Healthcare outcomes and special education eligibility in children with congenital CMV. PLoS One.

[38] Engman M.-L., Malm G., Engström L. (2008). Congenital CMV infection: prevalence in newborns and the impact on hearing deficit. Scand J Infect Dis.

[39] Forner G., Abate D., Mengoli C., Palu G., Gussetti N. (2015). High cytomegalovirus (CMV) DNAemia predicts CMV sequelae in asymptomatic congenitally infected newborns born to women with primary infection during pregnancy. J Infect Dis.

[40] Townsend C.L., Forsgren M., Ahlfors K., Ivarsson S.-A., Tookey P.A., Peckham C.S. (2013). Long-term outcomes of congenital cytomegalovirus infection in Sweden and the United Kingdom. Clin Infect Dis.

[41] Turriziani Colonna A., Buonsenso D., Pata D. (2020). Long-term clinical, audiological, visual, neurocognitive and behavioral outcome in children with symptomatic and asymptomatic congenital cytomegalovirus infection treated with valganciclovir. Front Med.

[42] Alarcon A., Martinez-Biarge M., Cabañas F., Hernanz A., Quero J., Garcia-Alix A. (2013). Clinical, biochemical, and neuroimaging findings predict long-term neurodevelopmental outcome in symptomatic congenital cytomegalovirus infection. J Pediatr.

[43] Dreher A.M., Arora N., Fowler K.B. (2014). Spectrum of disease and outcome in children with symptomatic congenital cytomegalovirus infection. J Pediatr.

[44] Fukushima S., Morioka I., Ohyama S. (2019). Prediction of poor neurological development in patients with symptomatic congenital cytomegalovirus diseases after oral valganciclovir treatment. Brain Dev.

[45] Kylat R.I., Kelly E.N., Ford-Jones E.L. (2006). Clinical findings and adverse outcome in neonates with symptomatic congenital cytomegalovirus (SCCMV) infection. Eur J Pediatr.

[46] Lanzieri T.M., Leung J., Caviness A.C. (2017). Long-term outcomes of children with symptomatic congenital cytomegalovirus disease. J Perinat.

[47] Pass R.F., Stagno S., Myers G.J., Alford C.A. (1980). Outcome of symptomatic congenital cytomegalovirus infection: results of long-term longitudinal follow-up. Pediatrics.

[48] Rossen J.L., Hindi A., Rahmani S., Bohnsack B.L. (2025). Incidence of ophthalmic manifestations in congenital cytomegalovirus (CMV). BMC Ophthalmol.

[49] Sterne J.A.C., Sutton A.J., Ioannidis J.P.A. (2011). Recommendations for examining and interpreting funnel plot asymmetry in meta-analyses of randomised controlled trials. BMJ.

[50] Maltezou P.-G., Kourlaba G., Kourkouni Ε. (2020). Maternal type of CMV infection and sequelae in infants with congenital CMV: systematic review and meta-analysis. J Clin Virol.

[51] Leruez-Ville M., Guilleminot T., Stirnemann J. (2020). Quantifying the burden of congenital cytomegalovirus infection with long-term sequelae in subsequent pregnancies of women seronegative at their first pregnancy. Clin Infect Dis.

[52] Bartlett A.W., McMullan B., Rawlinson W.D., Palasanthiran P. (2017). Hearing and neurodevelopmental outcomes for children with asymptomatic congenital cytomegalovirus infection: a systematic review. Rev Med Virol.

[53] Gabrani C., Mitsikas D., Giannakou K., Lamnisos D. (2023). Congenital cytomegalovirus infection and ophthalmological disorders: a systematic review. J Pediatr Ophthalmol Strabismus.

[54] World Health Organization (2021). Blindness and vision impairment. https://www.who.int/news-room/fact-sheets/detail/blindness-and-visual-impairment.

[55] Cannon M.J., Griffiths P.D., Aston V., Rawlinson W.D. (2014). Universal newborn screening for congenital CMV infection: what is the evidence of potential benefit?. Rev Med Virol.

[56] Miller J.H., Bardo D.M.E., Cornejo P. (2020). Neonatal neuroimaging. Semin Pediatr Neurol.

[57] Preiksaitis J.K., Hayden R.T., Tong Y. (2016). Are we there yet? Impact of the first international standard for cytomegalovirus DNA on the harmonization of results reported on plasma samples. Clin Infect Dis.

[58] Lo T.-H., Lin P.-H., Hsu W.-C. (2022). Prognostic determinants of hearing outcomes in children with congenital cytomegalovirus infection. Sci Rep.

[59] Smithers-Sheedy H., Khandaker G., Raynes-Greenow C. (2022). The long-term burden of congenital cytomegalovirus: hospitalisation and mortality in a population-based matched cohort study. Eur J Paediatr Neurol.

[60] Kirby T. (2016). Congenital Cytomegalovirus—A neglected health problem. Lancet Infect Dis.

[61] Gievers L.L., Holmes A.V., Loyal J. (2020). Ethical and public health implications of targeted screening for congenital cytomegalovirus. Pediatrics.

[62] Khalil A., Heath P.T., Jones C.E., Soe A., Ville Y.G. (2025). Congenital cytomegalovirus infection: update on screening, diagnosis and treatment. BJOG.

[63] Das R., Blázquez-Gamero D., Bernstein D.I. (2023). Safety, efficacy, and immunogenicity of a replication-defective human cytomegalovirus vaccine, V160, in cytomegalovirus-seronegative women: a double-blind, randomised, placebo-controlled, phase 2b trial. Lancet Infect Dis.

[64] Leruez-Ville M., Khalil A., Kagan K.O., Donner C., Lazzarotto T., Ville Y. (2019). Antenatal screening for cytomegalovirus infection: to know the chance, the chance to know. Lancet Child Adolesc Health.

[65] Schleiss M.R., Blázquez-Gamero D. (2025). Universal newborn screening for congenital cytomegalovirus infection. Lancet Child Adolesc Health.

[66] Solebo A., Emond A. (2019). Health for all children.

[67] Solebo A.L., Cumberland P.M., Rahi J.S. (2015). Whole-population vision screening in children aged 4–5 years to detect amblyopia. Lancet.

[68] World Health Organization (2022). Package of eye care interventions. https://iris.who.int/bitstream/handle/10665/354256/9789240048959-eng.pdf?sequence=1.

[69] Ibironke J.O., Friedman D.S., Repka M.X. (2011). Child development and refractive errors in preschool children. Optom Vis Sci.

[70] Sauer T., Lawrence L., Mayo-Ortega L., Oyama-Ganiko R., Schroeder S. (2018). Refractive error and ocular findings among infants and young children with severe problem behavior and developmental disabilities. J Ment Health Res Intellect Disabil.

[71] Levi D.M. (2020). Rethinking amblyopia 2020. Vision Res.

[72] Solebo A.L., Rahi J.S. (2023). Delayed diagnosis of congenital cataract in preterm infants: findings from the IoLunder2 cohort study. PLoS One.

