# Supplementary material for: Prevalence of ocular and visual abnormalities following symptomatic and asymptomatic congenital CMV infection: a systematic review and meta-analysis
Source: eClinicalMedicine. 2025 Sep 2;88:103443. doi: 10.1016/j.eclinm.2025.103443 (PMC12433491; doi:10.1016/j.eclinm.2025.103443)

Supplementary document

Contents

Search strategies 2

Data collection form 3

Figure S1: Critical appraisal summary 4

Figure S2: Ophthalmic assessments undertaken 5

Table S1. Subgroup comparisons for prevalence of visual impairment in populations with symptomatic cCMV 6

Table S2. Subgroup comparisons for prevalence of congenital ocular disorders in populations with symptomatic cCMV 7

Figure S3A-D. Subgroup meta-analyses of prevalence of congenital ocular disorders in populations with symptomatic cCMV 8

Figure S4: Forest plot of pooled prevalence of chorioretinal lesions in populations with symptomatic cCMV 10

Figure S5: Forest plot of pooled prevalence of optic nerve anomalies in populations with symptomatic cCMV 11

Figure S6: Forest plot of pooled prevalence of anterior segment dysgenesis / structural anomalies in populations with symptomatic cCMV 12

Figure S7A-D. Leave one out analyses 13

Table S3. Prevalence and type of congenital structural ocular disorders / ocular anomalies 15

Table S4. Prevalence and type of visual impairment 16

Table S5: Prevalence and type of other ocular and visual disorders 17

Figure S8A-D: Funnel plots assessing potential publication bias 18

Search strategies

<u>Medline</u>

1. exp Cytomegalovirus/
2. cmv.mp. [mp=title, abstract, original title, name of substance word, subject heading word, keyword heading word, protocol supplementary concept word, rare disease supplementary concept word, unique identifier]
3. cytomegalovirus*.mp. [mp=title, abstract, original title, name of substance word, subject heading word, keyword heading word, protocol supplementary concept word, rare disease supplementary concept word, unique identifier]
4. 1 or 2 or 3
5. congenital.mp. [mp=title, abstract, original title, name of substance word, subject heading word, keyword heading word, protocol supplementary concept word, rare disease supplementary concept word, unique identifier]
6. 4 and 5
7. limit 15 to human

<u>Embase</u>

1. Human cytomegalovirus/ or Cytomegalovirus antibody/ or Cytomegalovirus/
2. cytomegalovirus.tw.
3. CMV.tw.
4. 1 or 2 or 3
5. congenital
6. 4 and 5
7. 6 not ((exp animal/ or nonhuman/) not exp human/)

<u>Cochrane Database</u>

1. cytomegalovirus:ab,ti
2. congenital:ab,ti
3. #1 AND #2

<u>CINAHL Plus</u>

1. (MH "Cytomegalovirus Infections") OR "cmv"
2. “cytomegalovirus" OR (MH "Cytomegalovirus Infections")
3. hcmv
4. S1 OR S2 OR S3
5. (MH "Congenital") OR "congenital"
6. S4 and S5

Data collection form

Study ID	Text
sponsor	Text
Country	Text
Setting	Text
Authors name	Text
Institution	Text
Email	Text
Address	Text
Design	Text
Ophthalmic assessments undertaken	Text
Inclusion criteria	Text
Exclusion criteria	Text
OUTCOME: definition of visual disorders (eg, defn of blindness)	Text
OUTCOME: follow up time (age at final assessment, note median/average/minimum)	Text
OUTCOME: frequency of ocular disorders in Asymptomatic cCMV	Numerical
Details of ocular disorders in asymptomatic	Text
OUTCOME: frequency of ocular disorders in Symptomatic cCMV	Numerical
Details of ocular disorders in symptomatic	Text
OUTCOME: frequency of visual disorders in Asymptomatic cCMV	Numerical
Details of vision disorders in asymptomatic, number with VI	Text
OUTCOME: frequency of visual disorders in Symptomatic cCMV at follow up	Numerical
Details of vision disorders in symptomatic, number with VI	Text
OUTCOME: additional note on types of ocular disorders (ie ocular site) seen with prevalences / incidence	Text
Baseline Population ethnicity	Text
Baseline Population gender	Text
Baseline Population size	Numerical
Baseline Proportion born preterm (<37 gest weeks)	Numerical
Baseline Proportion with chorioretinitis at cCMV diagnosis	Numerical

Figure S1: Critical appraisal summary

Addressed?
Unclear
No
Yes

Study ID	Sample frame appropriate to address the target population?	Study participants sampled in an appropriate way?	Sample size adequate?	Study subjects and the setting described in detail?	Data analysed with sufficient coverage of identified sample?	Valid methods for identification of condition?	Condition measured in a standard, reliable way for all participants?	Appropriate statistical analysis?	Important subgroups / differences identified and accounted for?	Complete outcomes for populations, attrition managed appropriately?	Overall Risk of Bias
Capretti 2017	Yes	Yes	No	Yes	Yes	Yes	Yes	Yes	Yes	Yes	Low
Puhakka 2019	Yes	Yes	Yes	Yes	Yes	Yes	Yes	Yes	Yes	Yes	Low
Salomè 2023	Yes	Yes	Yes	Yes	Yes	Yes	Yes	Yes	Yes	Yes	Low
Auriti 2022	No	Yes	No	Yes	Yes	Yes	Yes	Yes	Yes	Yes	Moderate
Engman 2008	Yes	Yes	No	Yes	Yes	Yes	Yes	Unclear	No	Unclear	Moderate
Fukushima 2019	Yes	Yes	No	Yes	Yes	Unclear	Yes	Yes	Yes	Yes	Moderate
Jin 2017	Yes	Yes	Yes	Yes	No	Yes	No	Yes	Yes	Yes	Moderate
Jin 2019	Yes	Yes	Yes	Yes	No	Yes	No	Yes	Yes	Yes	Moderate
T Fahnehjelm 2015	No	Yes	No	No	Yes	Yes	Yes	Yes	Yes	Yes	Moderate
Townsend 2013	No	Yes	Yes	Yes	No	Unclear	No	Yes	Yes	Yes	Moderate
Kylat 2006	Yes	Yes	No	Yes	Unclear	Yes	Unclear	Yes	Yes	Yes	Moderate
Kaltorp 2014	No	Yes	No	No	Yes	Unclear	Unclear	Yes	Yes	Yes	High
Alarcon 2013	Yes	Yes	No	Yes	No	No	Unclear	Unclear	Unclear	Unclear	High
Dreher 2014	No	Yes	Yes	Yes	No	Unclear	No	No	Unclear	Unclear	High
Engman 2010	No	Yes	No	Yes	No	No	No	No	No	Yes	High
Forner 2015	Yes	Yes	No	Unclear	Unclear	Unclear	Unclear	Yes	Yes	Yes	High
Karimian 2016	Unclear	Yes	No	Yes	Unclear	Unclear	Unclear	Unclear	Yes	Yes	High
Korndewal 2017	Yes	Yes	Yes	Yes	No	Yes	Unclear	Yes	Unclear	Unclear	High
Lanzieri 2017	No	Yes	No	Yes	No	Yes	No	No	Yes	Yes	High
Lin 2020	No	Yes	No	Yes	No	No	No	Unclear	Yes	Yes	High
Marin 2016	No	Yes	No	Yes	No	Unclear	No	Unclear	Yes	Yes	High
Pass 1980	No	Unclear	No	Yes	Yes	Unclear	Yes	No	Unclear	Unclear	High
Stagno 1977	No	Unclear	No	No	No	Unclear	Unclear	Unclear	Unclear	Unclear	High
T Colonna 2020	Yes	No	No	No	No	No	No	No	Yes	Yes	High

Figure S2: Ophthalmic assessments undertaken

Study ID	Fundoscopy	Acuity	Orthoptic examination	Refraction	Anterior Segment	Adnexa	Unclear
Alarcon 2013							✓
Auriti 2022							
Capretti 2017							✓
Coats 2000	✓	✓	✓		✓		
Connolly 1992		✓					
Dreher 2014							✓
Engman 2008	✓	✓	✓	✓			
Engman 2010							✓
Forner 2015	✓						
Fukushima 2019							✓
Jin 2017	✓	✓	✓	✓	✓		
Jin 2019		✓					
Karimian 2016	✓						
Karltorp 2014	✓	✓					
Keymeulen 2023	✓	✓					
Korndewal 2017	✓						
Kylat 2006	✓	✓			✓		
Lanzieri 2017	✓	✓			✓		
Lin 2020	✓						
Marin 2016	✓						
Pass 1980							✓
Puhakka 2019	✓	✓	✓	✓	✓		
Saloma 2023							✓
Stagno 1977	✓	✓	✓	✓	✓		
Te Fahnehjelm 2015	✓	✓	✓	✓	✓	✓	
Townsend 2013							✓
Turriziani Colonna 2020	✓						
Visentin 2012	✓						✓

Table S1. Subgroup comparisons for prevalence of visual impairment in populations with symptomatic cCMV

Results of test of differences with subgroup Forest plot analyses

	**Test of group differences: Qb(1)**
Complete ocular assessment versus incomplete	2.38, p=0.12
Income level of study setting	All based in high income settings
Risk of bias moderate versus low	1.34, p=0.51
Study design prospective versus retrospective	0.00, p=0.96
Study period pre or = 2017 versus post 2017	1.14, p=0.29
Follow up duration less than or = 5 years versus more	1.22, p=0.27
Whole population versus identified through hearing impairment	3.31, p=0.07
Pre-term (<37 weeks) versus term	3.78, p=0.05

Table S2. Subgroup comparisons for prevalence of congenital ocular disorders in populations with symptomatic cCMV

Results of test of differences with subgroup Forest plot analyses

	**Test of group differences: Qb(1)**
Complete ocular assessment versus incomplete	0.70, p=0.40
Income level of study setting	-
Risk of bias moderate versus low	0.55, p=0.46
Study design prospective versus retrospective	0.68, p=0.41
Study period pre or = 2017 versus post 2017	43%, 95% CI 32-55 versus 10%, 3-24%<br>10.48, p<0.001
Follow up duration less than or = 5 years versus more	31%, 19-46%, versus 8%, 2 – 23%<br>5.45, p=0.02
Whole population versus identified through hearing impairment	12%, 5-29%, versus 38%, 22-58%<br>4.98, p=0.03
Pre-term (<37 weeks) versus term	34%, 17-56 versus 2%, 1-4%,<br>26.13, p<0.001

Figure S3A-D. Subgroup meta-analyses of prevalence of congenital ocular disorders in populations with symptomatic cCMV

Forest plot analyses for:

A. Study period pre or = 2017 versus post 2017

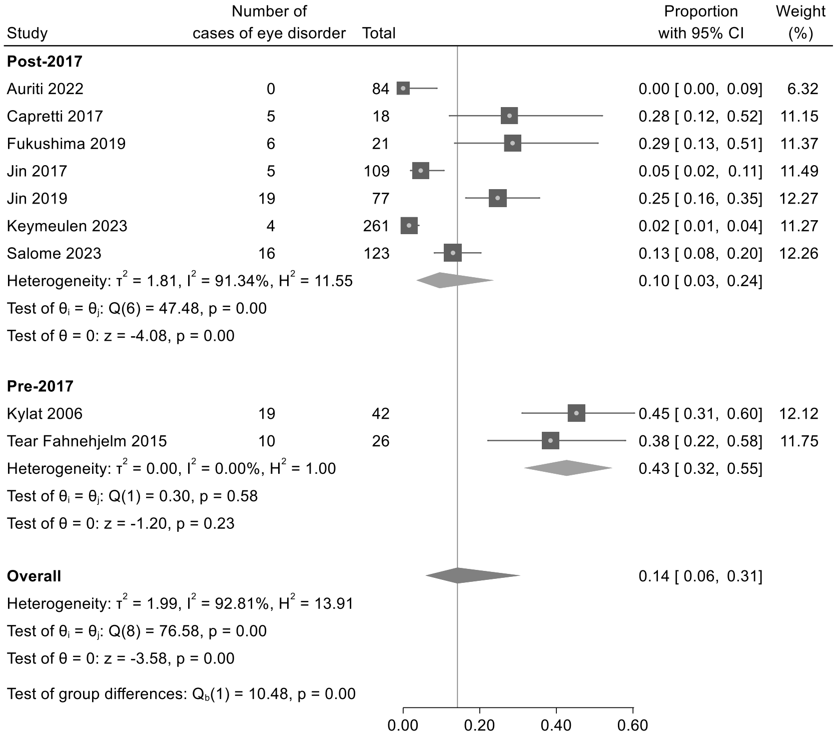

B. Follow up duration less than or = 5 years versus more

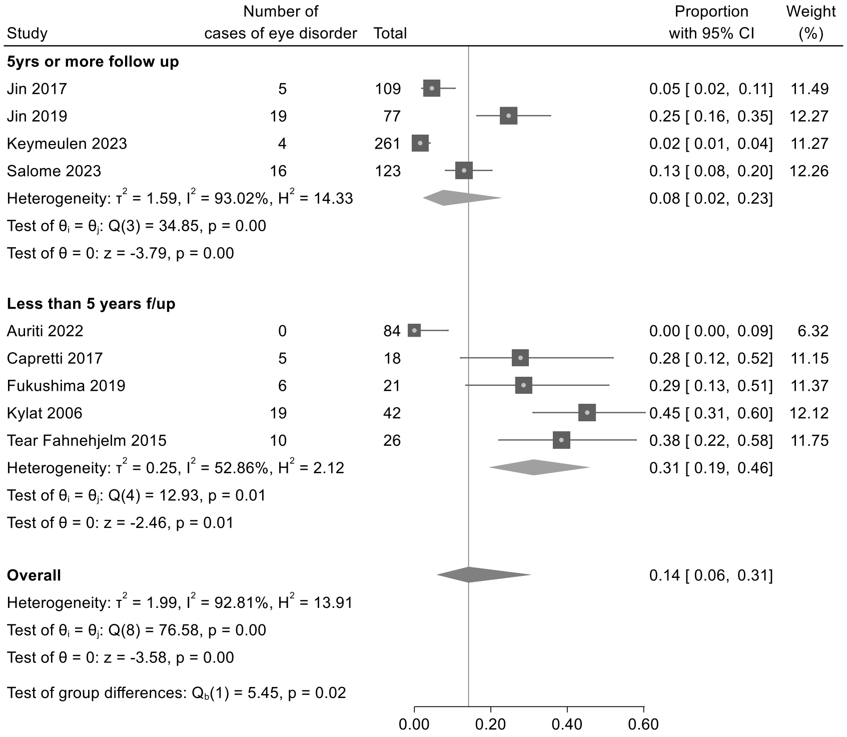

C. Whole population versus identified through hearing impairment

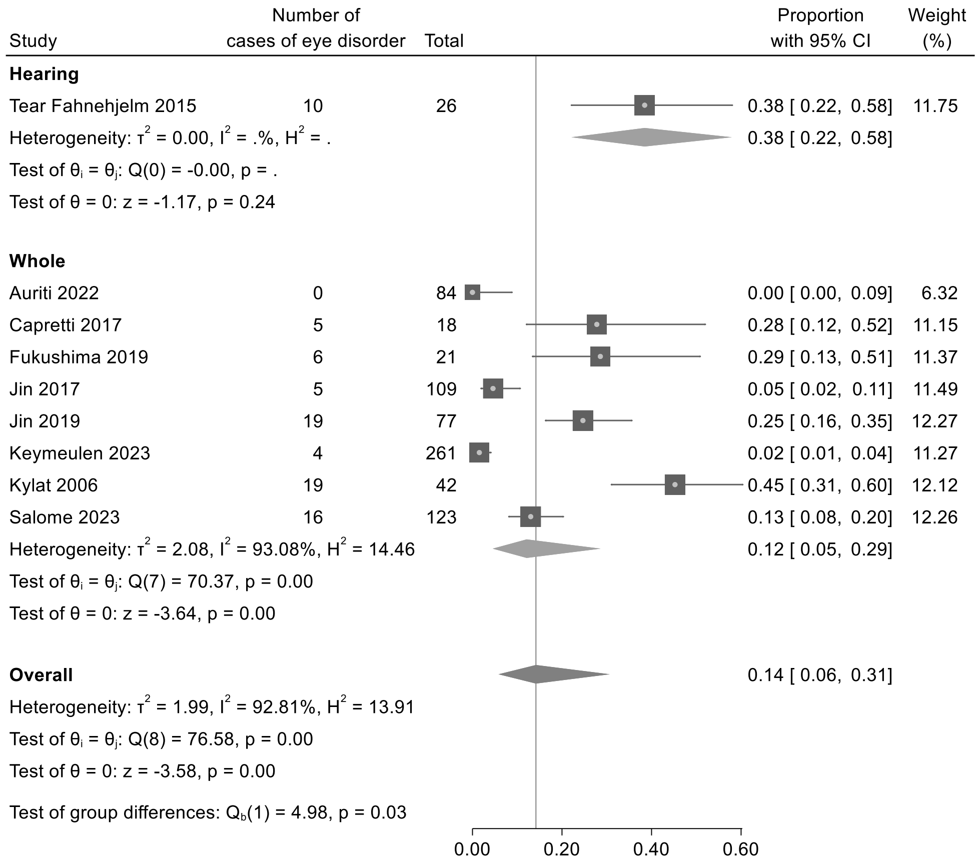

D. Pre-term (<37 weeks) versus term

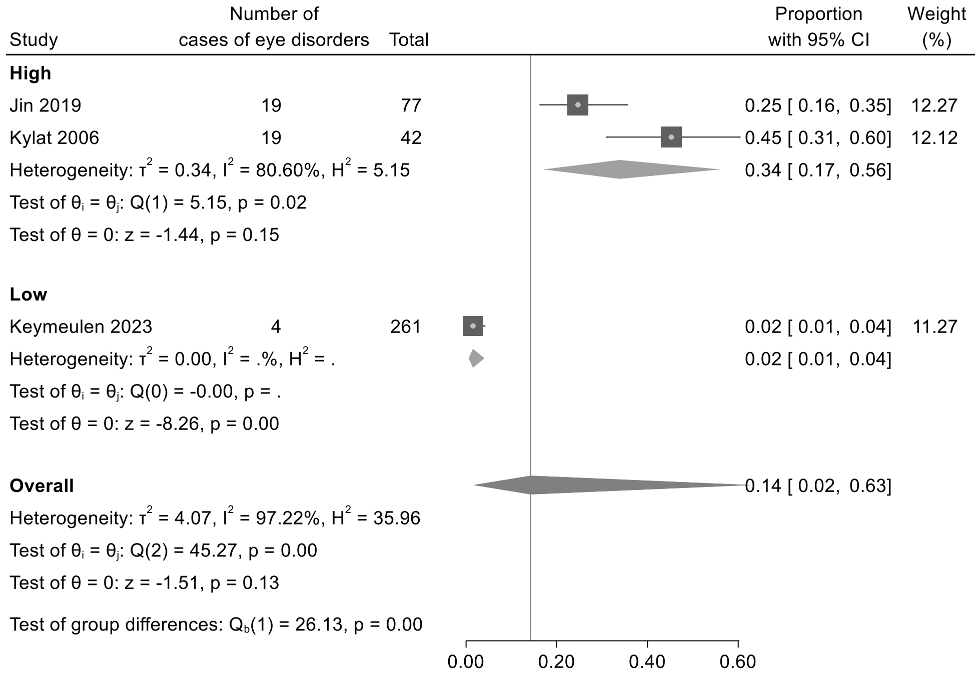

Figure S4: Forest plot of pooled prevalence of chorioretinal lesions in populations with symptomatic cCMV

Study	Number of cases of chorioretinal lesions	Total	Proportion with 95% CI	Weight (%)
Capretti 2017	5	18	0.28 [0.12, 0.52]	15.81
Fukushima 2019	6	21	0.29 [0.13, 0.51]	16.25
Jin 2019	19	77	0.25 [0.16, 0.35]	18.14
Keymeulen 2023	4	261	0.02 [0.01, 0.04]	16.04
Salome 2023	10	123	0.08 [0.04, 0.14]	17.65
Tear Fahnehjelm 2015	5	26	0.19 [0.08, 0.39]	16.11
**Overall**			0.14 [0.06, 0.30]	

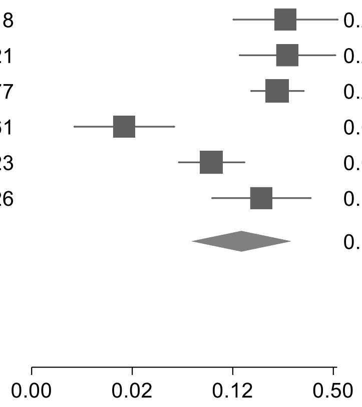

Heterogeneity: $\tau^2 = 1.34$, $I^2 = 89.08\%$, $H^2 = 9.16$

Test of $\theta_i = \theta_j$: Q(5) = 38.49, p = 0.00

Test of $\theta = 0$: z = -3.62, p = 0.00

Figure S5: Forest plot of pooled prevalence of optic nerve anomalies in populations with symptomatic cCMV

Study	Number of cases of chorioretinal lesions	Total	Proportion with 95% CI	Weight (%)
Capretti 2017	1	18	0.06 [0.01, 0.31]	15.35
Jin 2019	8	77	0.10 [0.05, 0.19]	37.86
Salome 2023	1	123	0.01 [0.00, 0.06]	15.87
Tear Fahnehjelm 2015	1	26	0.04 [0.01, 0.23]	15.54
Townsend 2013	1	19	0.05 [0.01, 0.29]	15.38
**Overall**			0.05 [0.02, 0.12]	

Heterogeneity: $\tau^2 = 0.49$, $I^2 = 42.28\%$, $H^2 = 1.73$

Test of $\theta_i = \theta_j$: Q(4) = 6.74, p = 0.15

Test of $\theta = 0$: z = -6.07, p = 0.00

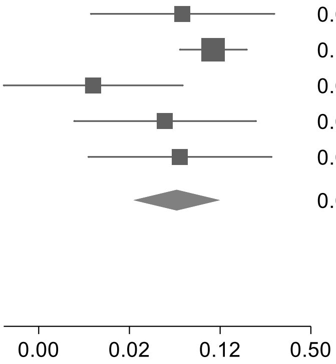

Figure S6: Forest plot of pooled prevalence of anterior segment dysgenesis / structural anomalies in populations with symptomatic cCMV

Study	Number of cases of anterior segment anomaly	Total	Proportion with 95% CI	Weight (%)
Jin 2019	5	77	0.06 [0.03, 0.15]	37.15
Kylat 2006	4	42	0.10 [0.04, 0.23]	33.27
Salome 2023	1	123	0.01 [0.00, 0.06]	14.97
Tear Fahnehjelm 2015	1	26	0.04 [0.01, 0.23]	14.62
**Overall**			0.05 [0.02, 0.11]	

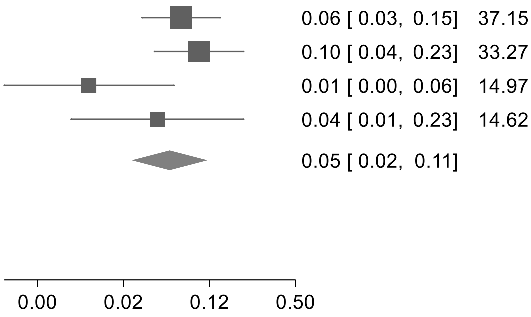

Heterogeneity: $\tau^2 = 0.32$, $I^2 = 41.66\%$, $H^2 = 1.71$

Test of $\theta_i = \theta_j$: Q(3) = 5.32, p = 0.15

Test of $\theta = 0$: z = -6.56, p = 0.00

Figure S7A-D. Leave-one-out analyses

A. Pooled prevalence of visual impairment in symptomatic cCMV: results of leave-one-out meta-analyses

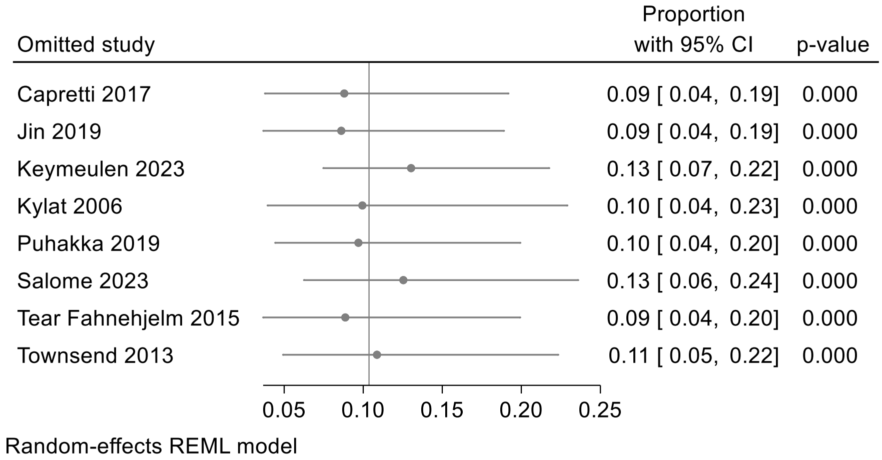

B. Pooled prevalence of ocular disorders in symptomatic cCMV: results of leave-one-out meta-analyses

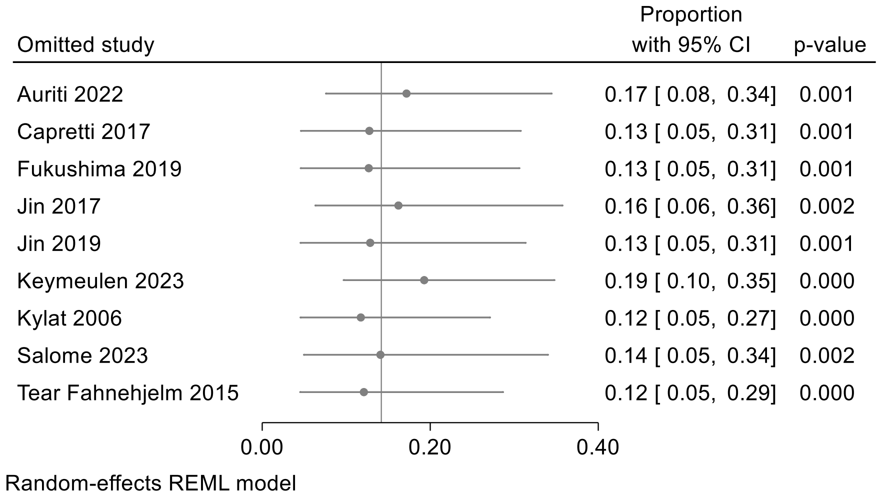

C. Pooled prevalence of visual impairment in asymptomatic cCMV: results of leave-one-out meta-analyses

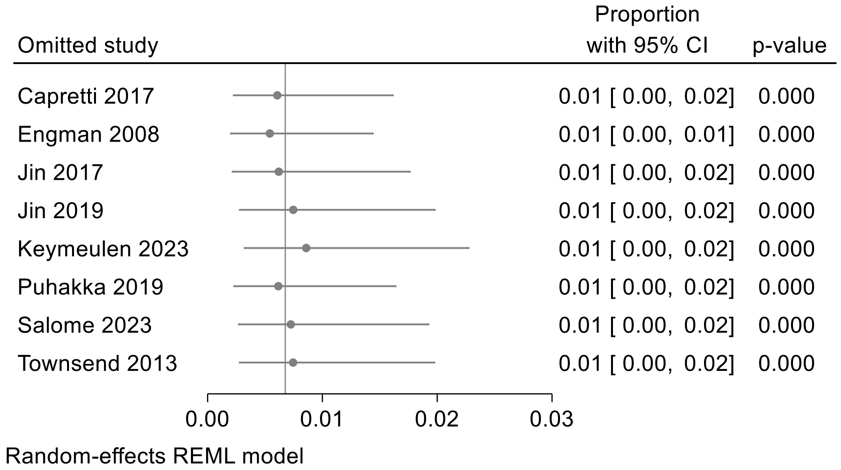

D. Pooled prevalence of ocular disorders in asymptomatic cCMV: results of leave-one-out meta-analyses

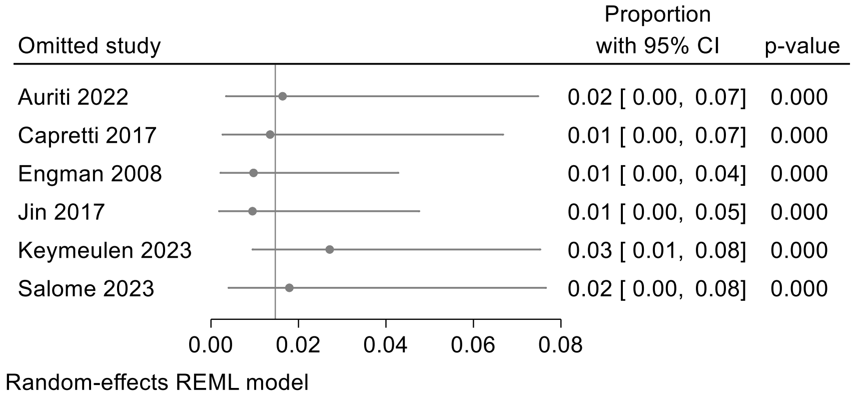

Table S3. Prevalence and type of congenital structural ocular disorders / ocular anomalies

	**Structural ocular disorder**	
Study	**Asymptomatic cCMV**	**Symptomatic cCMV**
Alarcon 2013*	-	Chorioretinitis: 1/24, 4%
Capretti 2017	0/30	Chorioretinal lesion(s): 5/18, 28%<br>Microphthalmia (bilateral): 1/18, 6%<br>Optic nerve hypoplasia: 1/18, 6%
Coats 2000	Macular lesion(s): 2/83, 2%	Macular lesion(s): 3/42, 7%<br>Periph retinal lesions: 6/42, 14%<br>Optic atrophy: 3/42, 7%
Dreher 2014*	-	Chorioretinitis: 23/166, 14%
Jin 2019*	-	Chorioretinitis: 19/77, 25%<br>Anterior segment abnormality / cataract: 7/77, 9%<br>Optic disc anomaly: 5/77, 6%<br>Vitreous haemorrhage: 7/77, 9%<br>Nystagmus: 11/77, 14%
Korndewal 2017	Optic atrophy: 1/107, 1%	Optic atrophy: 0/26
Kylat 2006*	-	Retinal lesions: 3/42, 7%<br>Anterior segment anomaly / cataract: 6/42, 14%
Lanzieri 2017*	-	Chorioretinitis: 19/76, 25%<br>Optic nerve atrophy: 9/76, 12%<br>Nystagmus: 11/76, 14%
Lin 2020	0/17	Chorioretinitis: 3/53, 6%
Pass 1980*	-	Chorioretinitis: 4/23, 17%<br>Optic atrophy: 3/23, 13%
Rochat 2024a	0/109	Optic atrophy: 7/77, 9%<br>Retinal detachment: 1/77, 1%
Rochat 2024b	Optic atrophy: 1/36, 3%<br>Retinal detachment: 0/36	Optic atrophy: 2/76, 3%<br>Retinal detachment: 1/76, 1%
Rossen 2025	0/20	Chorioretinitis: 2/52, 4%<br>Optic nerve hypoplasia, dysplasia, or atrophy: 12/52, 23%<br>Anterior segment anomaly / cataract: 4/52, 8%
Salome 2023	0/127	Chorioretinitis: 16/123, 13%<br>Microphthalmos: 1/123, 1%<br>Optic atrophy: 1/123, 1%
Stagno 1977	Nystagmus: 1/35, 3%	Chorioretinitis: 2/8, 25%<br>Optic atrophy: 2/8, 25%<br>Nystagmus: 2/8, 25%
Tear Fahnehjelm 2015*	-	Macular lesions: 5/26, 19%<br>Anterior ocular segment pathology: 1/26, 4%<br>Optic disc anomaly: 1/26, 4%
Townsend 2013*	-	Optic atrophy: 1/176, 1%

*Studies did not report on outcomes following asymptomatic cCMV

Table S4. Prevalence and type of visual impairment

Study ID	Prevalence of visual impairment (ie documentation of poor vision with both eyes open / poor vision in the better seeing eye)	
	**Asymptomatic cCMV**	**Symptomatic cCMV**
Jin 2019	0/160	Moderate or severe impairment: 15/77, 18%
Korndewal	Cerebral visual impairment (CVI): 2/107, 2%	Cerebral visual impairment (CVI): 0/26
Kylat 2006*	-	Blindness 4/42, 10%<br>Cortical visual impairment: 3/42, 7%
Lanzieri 2017*	-	Mild/moderate visual impairment: 8/76, 11%<br>Low vision: 4/76, 5%<br>Legal blindness: 10/76, 13%
Rochat 2024a	0/109	Severe visual impairment: 10/77, 10%<br>Cortical visual impairment: 11/77, 14%
Rochat 2024b	Severe visual impairment: 1/36, 3%<br>Cortical visual impairment: 0/36	Severe visual impairment: 4/76, 5%<br>Cortical visual impairment: 7/76, 9%
Rossen 2025	0/20	Cortical visual impairment: 2/52, 4%

*Studies did not report on outcomes following asymptomatic cCMV

Table S5: Prevalence and type of other ocular and visual disorders

Study ID	Prevalence of other eye and vision disorders	
	**Asymptomatic cCMV**	**Symptomatic cCMV**
Capretti 2017*	-	Strabismus: 1/18, 6%
Jin 2019*	-	Nystagmus: 11/77, 14%<br>Strabismus: 18/77, 23%<br>Astigmatism: 22/77, 29%
Kylat 2006	Strabismus: 2/107, 2%<br>Amblyopia: 3/107, 3%<br>Refractive error: 9/107, 8%	Strabismus: : 0/26<br>Amblyopia: 0/26<br>Refractive error: 4/26, 15%
Stagno 1977	Strabismus: 0/35<br>Nystagmus: 1/35, 3%<br>Amblyopia: 0/35<br>Astigmatism: 0/35	Strabismus: 19/76, 25%<br>Nystagmus: 11/76, 14%<br>Amblyopia: 3/76. 4%<br>Astigmatism: 22/76, 29%
Tear Fahnehjelm 2015	Nystagmus: 1/35, 3%<br>Strabismus: 1/35, 3%	Nystagmus: 2/8, 25%<br>Strabismus: 3/8, 38%
Townsend 2013*		Strabismus: 5/20, 25%

*Studies did not report on frequency of individual disorders following asymptomatic cCMV

Figure S8A-D: Funnel plots assessing potential publication bias

A. Studies on prevalence of visual impairment in symptomatic cCMV

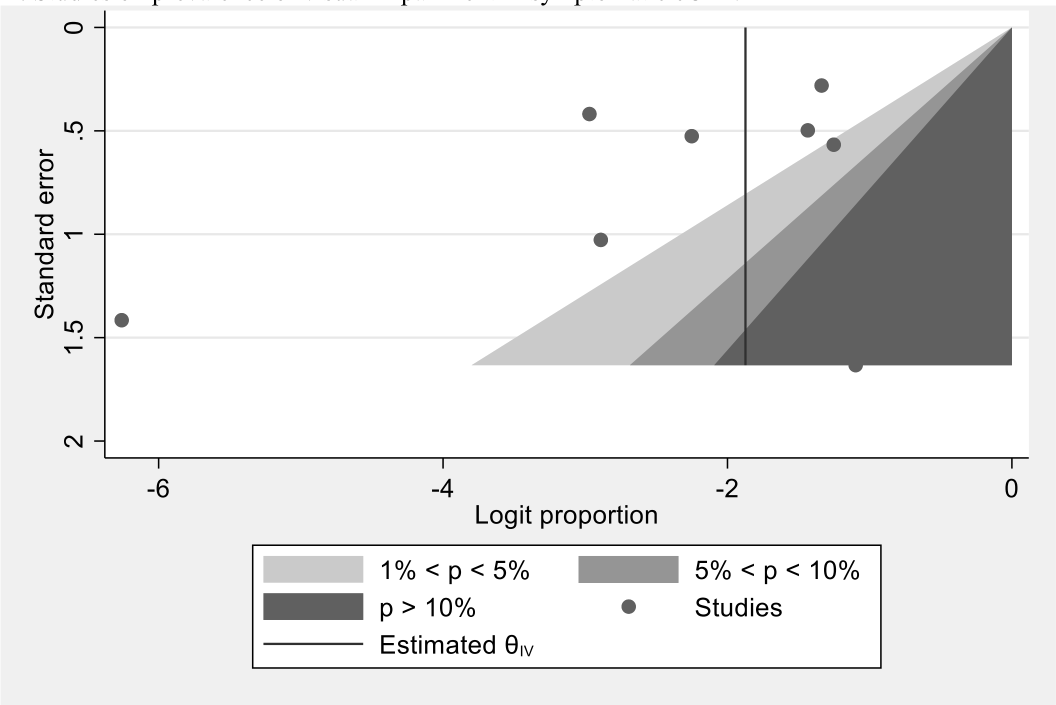

B. Studies on prevalence of ocular disorders in symptomatic cCMV

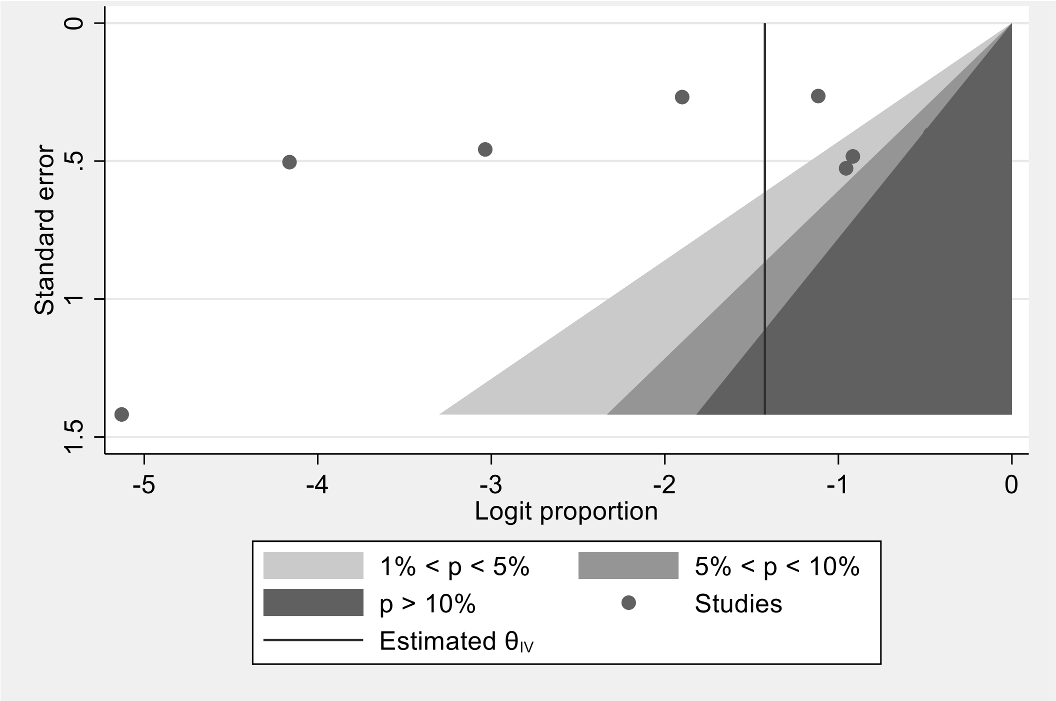

C. Studies on prevalence of visual impairment in asymptomatic cCMV

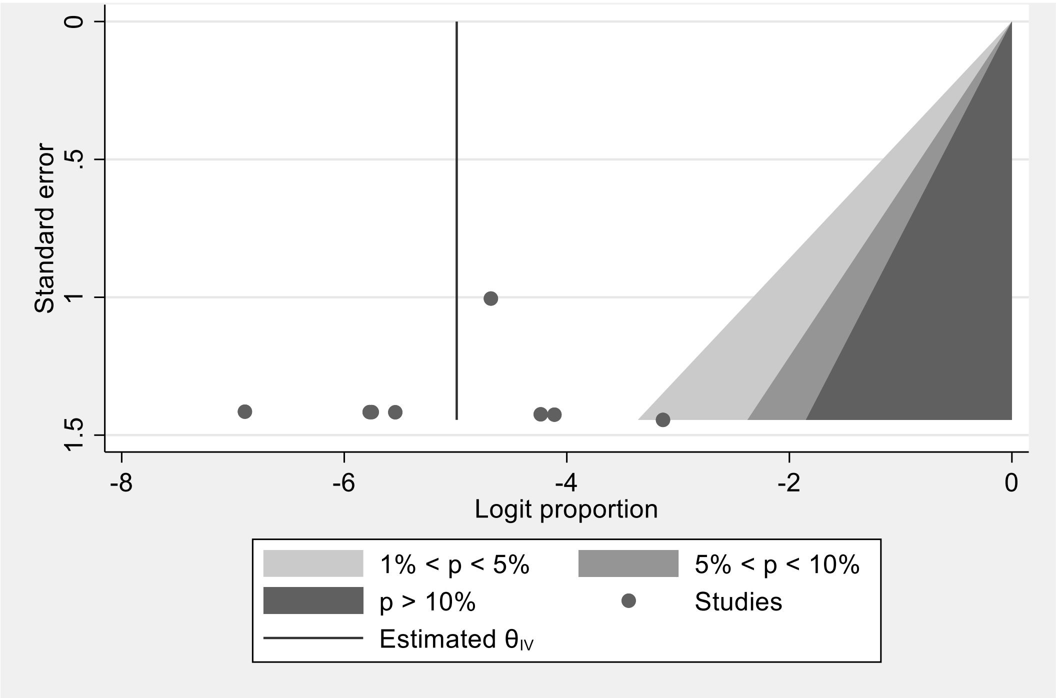

D. Studies on prevalence of ocular disorders in asymptomatic cCMV

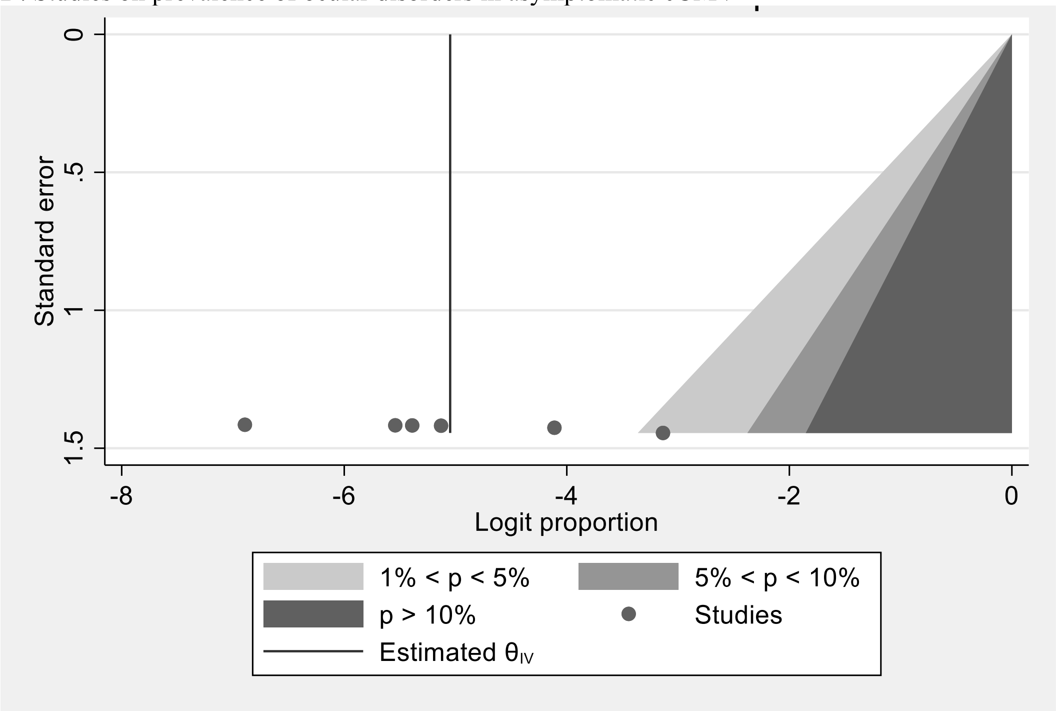

Supplement: Supplementary Figures and Tables [file mmc1.pdf]
